# GIWT-YOLO: an efficient multi-scale framework for real-time Scolytinae pests detection

**DOI:** 10.3389/finsc.2025.1635439

**Published:** 2025-09-26

**Authors:** Jingwei Liu, Yongke Li, Lei Wang, Yunjie Zhao, Bowen Mao, Pengying Wang

**Affiliations:** ^1^ College of Computer and Information Engineering, Xinjiang Agricultural University, Urumqi, China; ^2^ Xinjiang Agricultural Informatization Engineering Technology Research Center, Xinjiang Agricultural University, Urumqi, China; ^3^ Research Center for Intelligent Agriculture, Ministry of Education Engineering, Urumqi, China

**Keywords:** pest detection, Scolytinae pests, lightweight model, YOLOv11s, multi-scale convolutional, effective receptive field, SE attention mechanism

## Abstract

The broad range of Scolytinae pests sizes and their subtle visual similarities, especially in smaller species, continue to challenge the accuracy of mainstream object detection models. To address these challenges, this paper proposes GIWT-YOLO, a lightweight detection model based on YOLOv11s, specifically tailored for Scolytinae pests detection. (1) We designed a lightweight multi-scale convolution module, GIConv, to improve the model’s ability to extract features at different pest scales. This module enhances the accuracy of small-object detection while reducing the computational cost and parameter complexity of the backbone. (2) The WTConv module inspired by wavelet transform is introduced into the backbone. This enlarges the effective receptive field and improves the model’s ability to distinguish pests with similar textures. (3) An SE attention mechanism is incorporated between the Neck and Head to enhance the model’s focus on key feature regions. Experimental results show that GIWT-YOLO achieves 84.7% in Precision, 88.7% in mAP@50, and 63.4% in mAP@50~95, which are improvements of 2.2%, 4.0%, and 3.1%, respectively, compared to the baseline YOLOv11s. Additionally, the model’s parameters and GFLOPs are reduced by 11.3% and 13.4%, respectively. Our proposed model surpasses the state-of-the-art (SOTA) performance in small-sized pest detection while maintaining a lightweight architecture, and its generalization ability has been validated on other public datasets. Our model provides an efficient solution for detecting Scolytinae pests. In future work, we plan to collect additional images of various pest species to expand the dataset, further enhancing the model’s applicability to a wider range of pest detection scenarios.

## 1 Introduction

As one of the largest forestry countries in the world, China has experienced a sharp increase in harmful organisms in recent years due to the impact of pests and diseases, resulting in significant economic losses and posing severe challenges to forestry development (1). However, due to the wide variety of pests, the low accuracy of pest identification has been plaguing personnel (2). The traditional method is mainly identified by professionals, which has a huge workload, low efficiency. Additionally, it lacks real-time monitoring of pest outbreaks, making it difficult to implement preventive strategies in a timely manner. Therefore, timely and accurate detection and identification of forest pests are crucial for effective forest management (3).

The subfamily Scolytinae is among the most destructive forest pests, widely distributed across forest ecosystems worldwide, and poses serious threats particularly to coniferous species such as pine and spruce (4). With the development of deep learning technology, it has become a more effective scheme to use convolutional neural network technology to realize the automatic detection of forestry pests. Applying it to pest identification can not only significantly improve recognition accuracy and processing efficiency, but also enable the automated analysis of large volumes of trap images, thereby overcoming the limitations of manual identification in terms of time, labor, and accuracy. However, due to the wide variety of pests and large differences in morphology and size, there are problems such as false detection, and overlapping detection.

Traditional pest identification methods primarily rely on image processing and feature engineering, combined with machine learning algorithms for pest classification. Wang et al. (5) proposed a whitefly counting algorithm based on K-means clustering and ellipse fitting. Deng et al. (6) extended the HMAX model, utilizing Scale-Invariant Feature Transform (SIFT) and Non-negative Sparse Coding (NNSC) to extract features, followed by the use of SVM for pest identification. Ma et al. (7) introduced an SVM classifier based on a combination of global features and HOG features for pest classification. Yang et al. (8) proposed an image recognition algorithm for pests on greenhouse whitefly and thrips trap boards, using Prewitt and Canny edge detection operators for segmentation, followed by SVM. However, this method is for a single pest species and does not consider pest detection in complex environments. Peng and Jinlan (9) proposed a small green leafhopper recognition method based on PCA-LDA-SVM. Despite progress in feature extraction, these methods still struggle to meet practical requirements. Machine learning-based vision algorithms face issues in complex environments, such as low recognition accuracy, slow inference speed, and poor robustness. Furthermore, they tend to rely heavily on the color of static pests for identification, over-depend on sample-specific features, and exhibit poor generalization capability.

In recent years, deep convolutional neural networks have gained widespread attention in the field of forestry pest detection. YOLO (You Only Look Once) (10–16) series algorithms have achieved an effective balance between detection accuracy and computational complexity, garnering significant interest and widespread application among researchers. Zhong et al. (17) proposed a method to count the number of flying insects in images using the YOLO model for object detection, followed by further classification through SVM. However, the study primarily focused on simple backgrounds and lacked research on insect recognition in more complex environments. Bai et al. (18) proposed a MOG2-YOLOv4 detection model for East Asian migratory locusts, which effectively solved the problem of low recognition accuracy caused by high speed movement and occlusion of East Asian migratory locusts. Wang (19) used an improved ALexNet model to identify agricultural pests, which effectively accelerated the inference speed of the model. Zhang et al. (20) improved the YOLOv3 model and combined Spatial Pyramid Pooling (SPP) with it to improve the accuracy of small-size pests. However, the model’s complexity was not considered, which limited its applicability for deployment on edge devices. Bhatt et al. (21) used YOLOv3 model for object detection in tea garden pest images, and the mAP reached 86% while maintaining the same inference performance. Liu and Wang (22) improved the YOLOv3 model by adding Spatial Pyramid Pooling (SPP) and utilizing multi-scale techniques to better capture tomato pest features, achieving a detection accuracy of 92.39%. Wen et al. (23) enhanced the YOLOv4 model on the Pest24 dataset and proposed the PEST-YOLO detection method for detecting multiple types of dense, tiny pests. However, the focus was primarily on improving missed detection issues, neglecting the balance between detection accuracy and mAP. Zhongzhu et al. (24) insert a Global Attention Upsampling (GAU) module into the output layer of the backbone network. This module uses global information from high-level features to help the model to extract features from complex backgrounds, improving recognition accuracy. However, it still suffers from high false detection rates. Yuan et al. (25) added a convolutional attention module to improve the feature expression of tea garden pests. Jiang and Yang (26) proposed a lightweight pest detection algorithm based on YOLOv8n (Bm-YOLO), designed the MCCA attention mechanism module to integrate with C2f, and performed well on the IP102 dataset, but the recognition accuracy is still insufficient in complex environments. There are some problems such as false detection, missed detection, and overlapping detection. Although the above studies have achieved certain improvements in detection accuracy, the adopted models are computationally complex and lack of lightweight optimization, which limits their application on edge devices.

Due to the limited lightweight capability of existing models in pest detection tasks, deploying them on edge devices is challenging. In addition, the similarity of features among Scolytinae pests makes feature extraction difficult. This is especially true for small pests, whose tiny size and fuzzy texture make detection harder. To address the above issues, we propose GIWT-YOLO, an improved object detection model specifically designed for tiny pests. Its architecture is based on YOLOv11s (13), a recent framework introduced by Khanam and Hussain in 2024, which serves as a strong and modern baseline. The following improvements are made in this paper:

1. We proposed a lightweight model, GIWT-YOLO, for detecting small and visually similar pest targets. The model improves detection accuracy for small pests and those with similar appearances.
2. We proposed GIConv, a convolution module suitable for multi-scale pest detection, to replace the standard convolution in the YOLOv11s backbone. This module improves the model’s ability to detect pests at different scales while making the backbone network more lightweight.
3. In the C3K2 structure, the WTConv module, inspired by wavelet transform, is introduced to construct the C3K2_WT structure. This modification enlarges the effective receptive field, improving the model’s ability to distinguish pests with similar textures. At the same time, it reduces model complexity and maintains inference efficiency.
4. Considering the high morphological similarity among different pest species, this paper introduces the SE attention mechanism module between the Neck and Head networks. This module enhances the model’s focus on key feature regions, enabling it to more accurately capture differences between pest categories and improve its ability to differentiate similar pests.

The structure of this paper is as follows: Section 2 introduces the dataset acquisition and the GIWT-YOLO model architecture, with detailed explanations of the improvements made to each module. Section 3 covers the experimental design and result analysis. Finally, Sections 4 and 5 provide the discussion and conclusion.

## 2 Materials and methods

### 2.1 Dataset introduction

**Figure 1:**
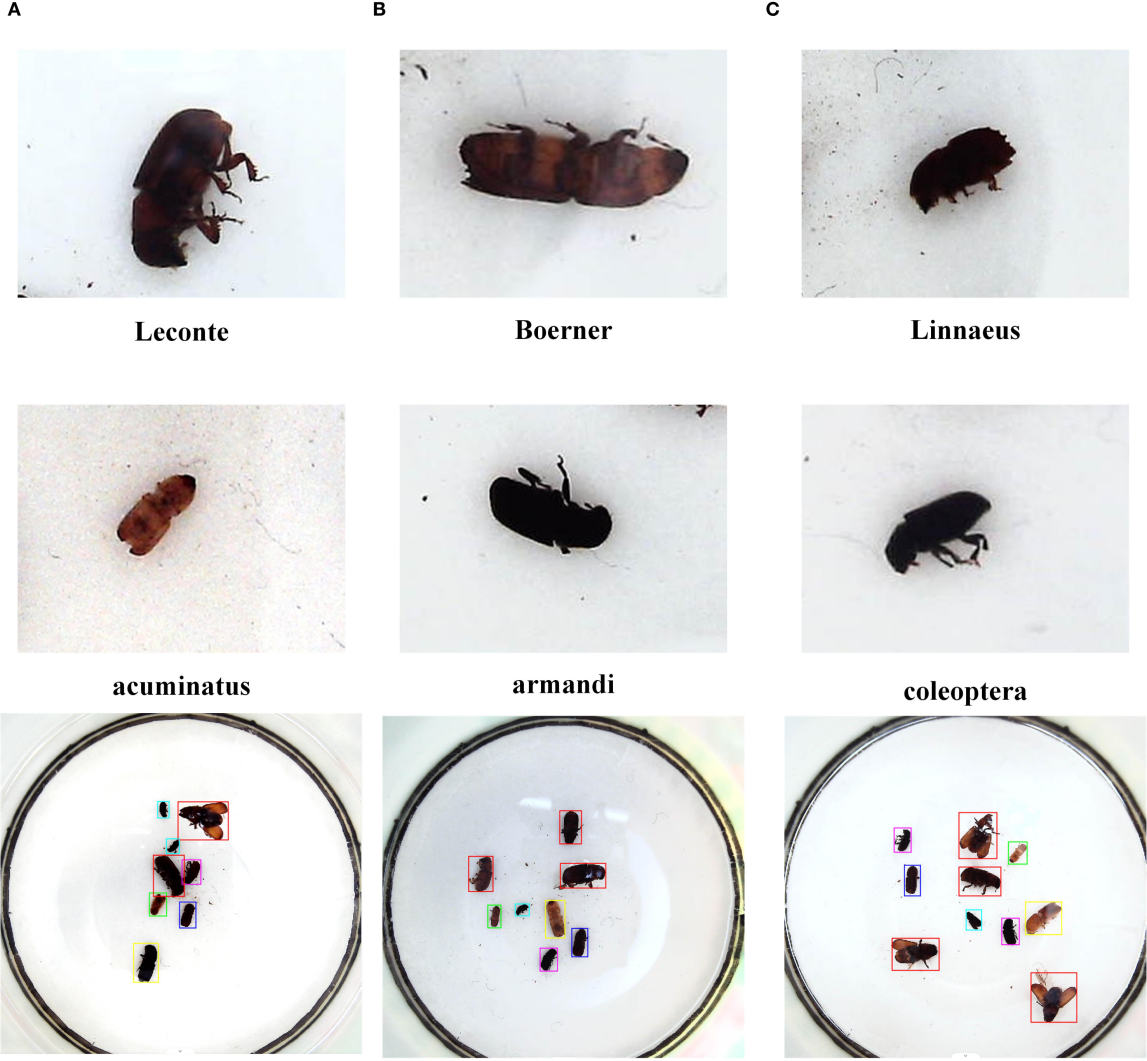
Data sample image.

The dataset utilized in this research was sourced from the Baidu AI Studio platform, an open online environment powered by PaddlePaddle, which facilitates data sharing, collaborative development, and participation in machine learning challenges. The dataset used in this study is the Scolytinae pests dataset provided by Beijing Forestry University (4) (https://aistudio.baidu.com/datasetdetail/51399, accessed on 15 January 2025). The dataset contains 2,183 image samples. Some sample images from the dataset are shown in **Figure 1**. These images include six insect categories: Boerner, Leconte, Linnaeus, Acuminatus, Armandi, and Coleoptera. In this study, the pest images are manually annotated using the LabelImg tool, with each image’s annotations stored in an Extensible Markup Language (XML) (27) file following the VOC format.

To ensure the effectiveness of model training, the original dataset of 2,183 image samples was divided into a training set (1,693 images), a validation set (245 images), and a test set (245 images). The pest samples in this dataset exhibit significant differences in body size, and the images were captured under varying lighting conditions. As illustrated in **Figures 1A–C**, these images show different levels of illumination. Considering that data diversity can enhance training stability, this study adopts the Mixup (28) and Mosaic (10) data augmentation strategies, without employing additional offline augmentation techniques. This approach enhances data diversity and improves the generalization ability of the model, while avoiding the potential noise introduced by excessive augmentation.

### 2.2 GIWT-YOLO pest detection model

**Figure 2:**
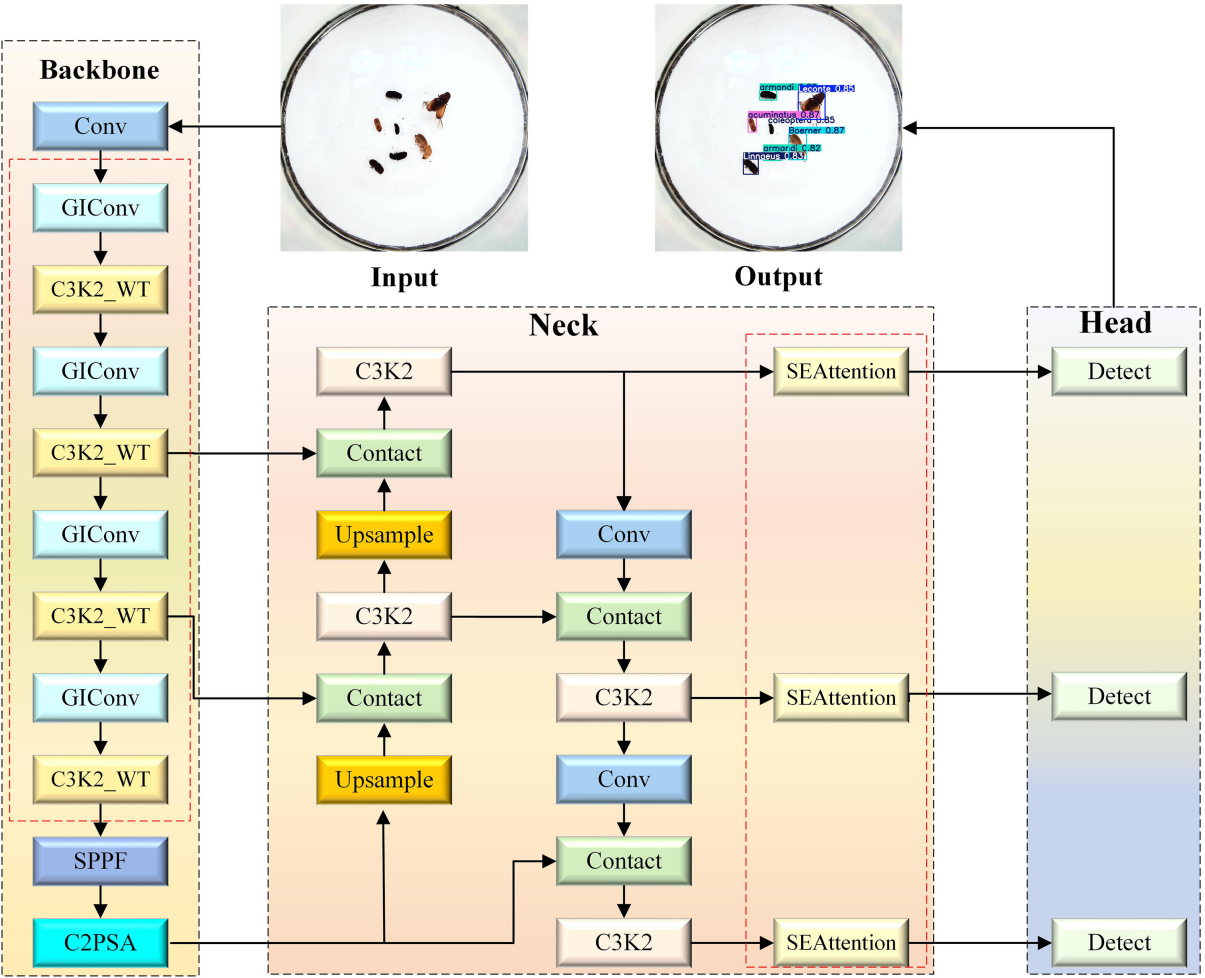
The proposed GIWT-YOLO algorithm model. The red dashed line represents the added improvement module.

The YOLOv11s (13) model has achieved significant improvements in both inference performance and accuracy. However, it still involves high computational complexity and performs poorly in detecting small objects. To solve this problem, this study proposes an improved lightweight GIWT-YOLO model based on YOLOv11s, and its model structure is shown in **Figure 2**. In order to achieve a lightweight network and high pest detection accuracy, GIWT-YOLO mainly makes the following improvements. First, the GIConv module is introduced to replace the ordinary convolution in the YOLOv11s backbone. This enhances the model’s ability to detect pests at different scales, especially improving recognition accuracy for small-object pests. It reduces the amount of computation and the number of parameters at the same time. Secondly, in the C3K2 structure, the WTConv module is introduced to construct the C3K2_WT structure. This modification increases the effective receptive field, thereby enhancing the model’s ability to detect pests with similar feature textures. Finally, Considering the high morphological similarity among different pest species, this paper introduces an SE attention mechanism module between the Neck and Head networks. This module enhances the model’s focus on key feature regions, enabling it to more accurately capture differences between pest categories and improve its ability to differentiate similar pests.

### 2.3 GIConv module design

**Figure 3:**
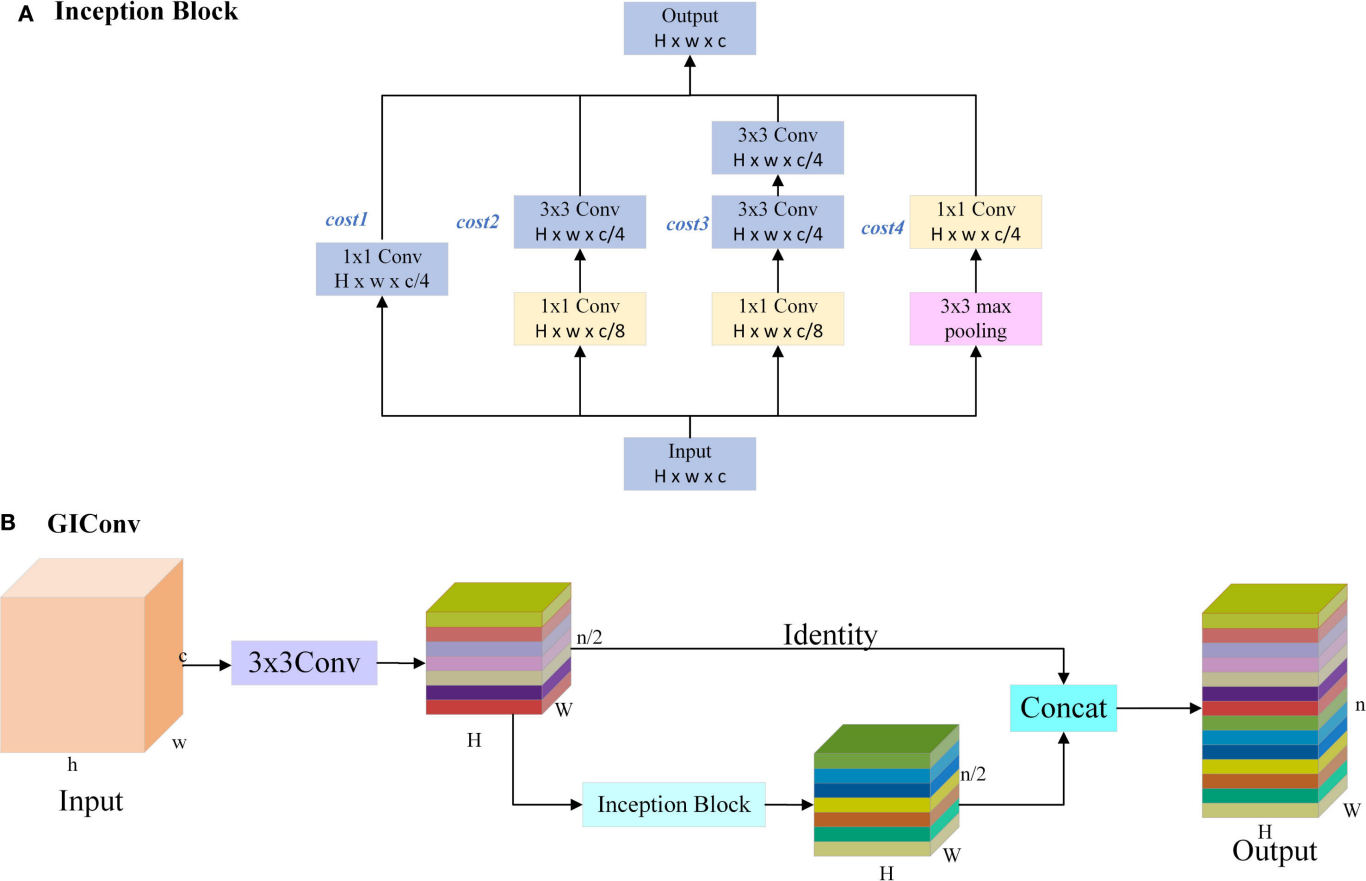
The structure of the GIConv module. **(A)** shows a parallel multi-branch structure with multi-scale convolutions; **(B)** shows the structure of GIConv.

As the pests in the Scolytinae pests dataset show more obvious body size differences, it is difficult for a single-scale convolution to take into account the feature extraction of pests of different sizes. Inspired by the Inception (29) module and GhostConv (30), we propose Ghost Inception Convolution(GIConv), which eliminates the use of simple linear operation. Firstly, a part of the core feature map is obtained using the 3x3 convolution module. Then, the Inception module (shown in **Figure 3A**) is utilized to obtain a multi-scale feature map. Finally, the core feature map and multi-scale feature map are spliced by Concat to finally obtain a multi-scale feature map. The convolution makes full use of the advantages of multi-scale depth convolution. It effectively solves the problem of missing feature information caused by the difference in pest body size by paralleling convolution layers of different scales. As a result, the model can extract global information from large object pests. At the same time, it pays more attention to the detailed features of tiny pests, thus improving detection accuracy. In addition, compared with ordinary convolution, the GIConv module significantly reduces the computational volume, and improves the computational efficiency and overall performance of the model. Its structure is shown in **Figure 3B**.

The amount of computation for each parallel path of the Inception module and the total computation cost, which is calculated as follows:


(1) $$\cos t_{1}=\frac{1}{4}\times H\times w\times c^{2}$$

(2) $$\cos t_{2}=H\times w\times c\times \frac{c}{8}+3\times 3\times \frac{c}{8}\times H\times w\times \frac{c}{4}=\frac{13}{32}\times H\times w\times c^{2}$$

(3) $$\cos t_{3}=H\times w\times c\times \frac{c}{8}+3\times 3\times \frac{c}{8}\times H\times w\times \frac{c}{4}+3\times 3\times \frac{c}{4}\times H\times w\times \frac{c}{4}=\frac{31}{32}\times H\times w\times c^{2}$$

(4) $$\cos t_{4}=\frac{1}{4}\times H\times w\times c^{2}$$

(5) $$\cos t=\cos t_{1}+\cos t_{2}+\cos t_{3}+\cos t_{4}=\frac{15}{8}\times H\times w\times c^{2}$$

In Equations 1-5, *H, w, c* are the height, width and number of channels of the input and output feature maps; The computation amount of the four parallel paths is: $\cos t_{1},\cos t_{2},\cos t_{3},\cos t_{4}$ , and the total computation amount of the final Inception module is:*cost*.

The amount of computation required for ordinary convolution in YOLO11s is:


(6) costa=H×W×n×k×k×c

If the ordinary convolution in YOLO11s is replaced by the GIConv module, the computational cost of the module is as follows:


(7) $$\cos tb=H\times W\times \frac{n}{2}\times k\times k\times c+\cos t=H\times W\times \frac{n}{2}\times k\times k\times c+\frac{15}{8}\times H\times W\times {\left(\frac{n}{2}\right)}^{2}$$

(8) $$S=\frac{\cos tb}{\cos ta}=\frac{1}{2}+\frac{15n}{32\times k^{2}\times c}$$

In Equations 6, 7, and 8, *h, w, c* are the height, width, and number of channels of the input feature maps; *H, W, n* are the height, width, and number of channels of the output feature maps; and S is the ratio of the computation amount of the GIConv module to the computation amount of the ordinary convolution. In this study, k=3 and n=2c, S is approximately equal to 0.6, so the computation of GIConv module is reduced to 60% of the ordinary convolution module.

### 2.4 C3K2_WT module

**Figure 4:**
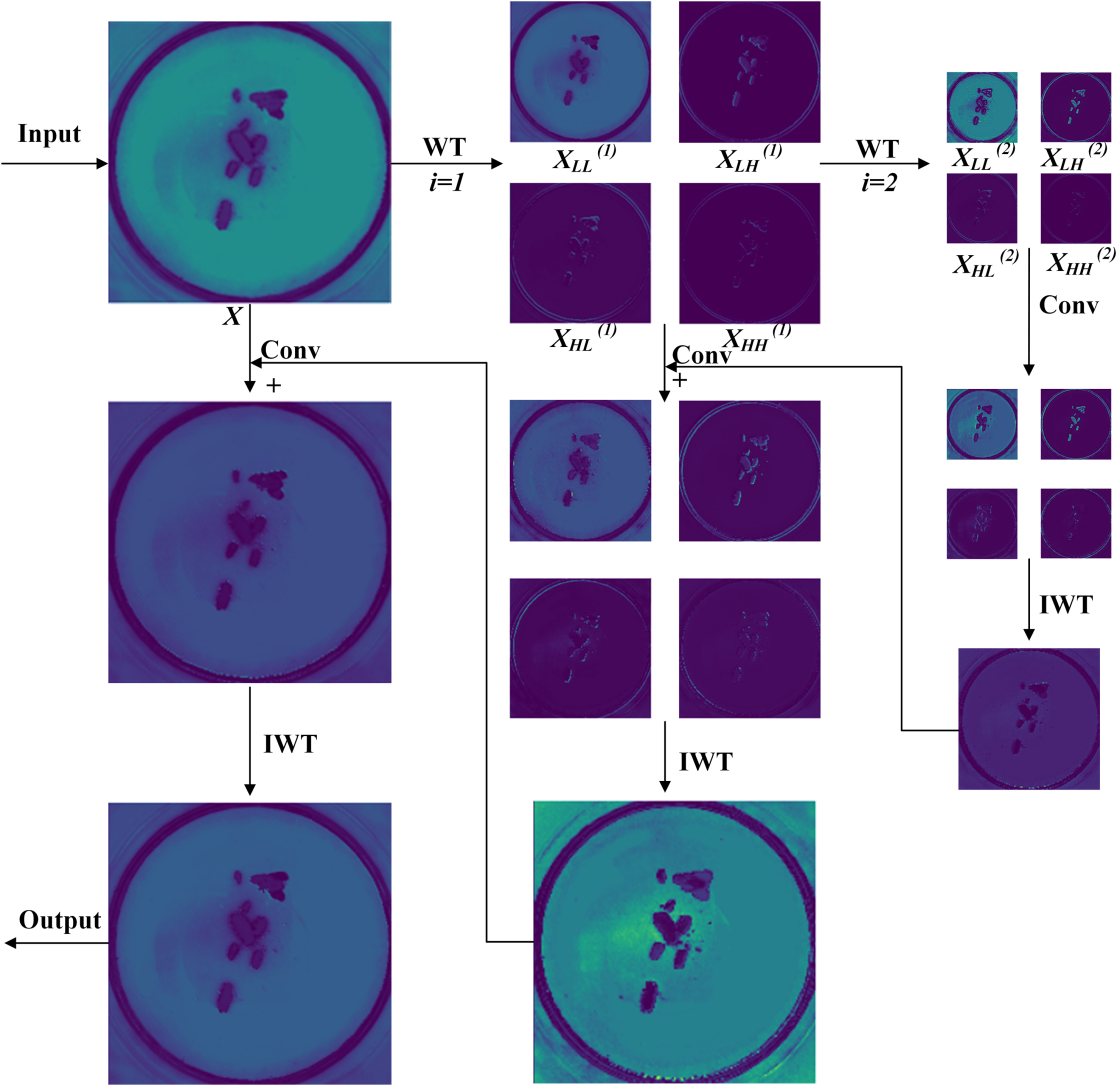
WTConv module structure. Wavelet Transform (WT) Principle, *X* is the input feature map, $X_{LL}$ is the low-frequency component, and $X_{LH},X_{HL},X_{HH}$ is the horizontal, vertical and diagonal high-frequency components. Among them, when *i* = 0, $X_{LL}^{\left(0\right)}=X$ , *i* represents the current level. "+" represents the Concat operation. IWT represents the Inverse Wavelet Transform operation.

Due to the similar texture and color of some pests in the data samples. It is difficult for the C3K2 module to extract enough key features, which affects the classification and detection accuracy of the pests. Therefore, a large size convolutional kernel is used to expand the receptive field to capture more detailed features. However, the use of larger convolutional kernels results in a quadratic growth in computational complexity, which presents a significant challenge for achieving model lightweighting. To address this issue, WTConv (31) introduces the Wavelet Transform (WT). It uses a set of small-sized convolutional modules, with each convolution focusing on a different frequency bands of the input, resulting in an increasingly larger receptive field. The structural diagram of WTConv is shown in **Figure 4**. Notably, the number of parameters in WTConv increases logarithmically with the expansion of the receptive field. Compared with standard convolutional methods, WTConv achieves a larger receptive field while effectively avoiding the problem of excessive parameter growth.

**Figure 5:**
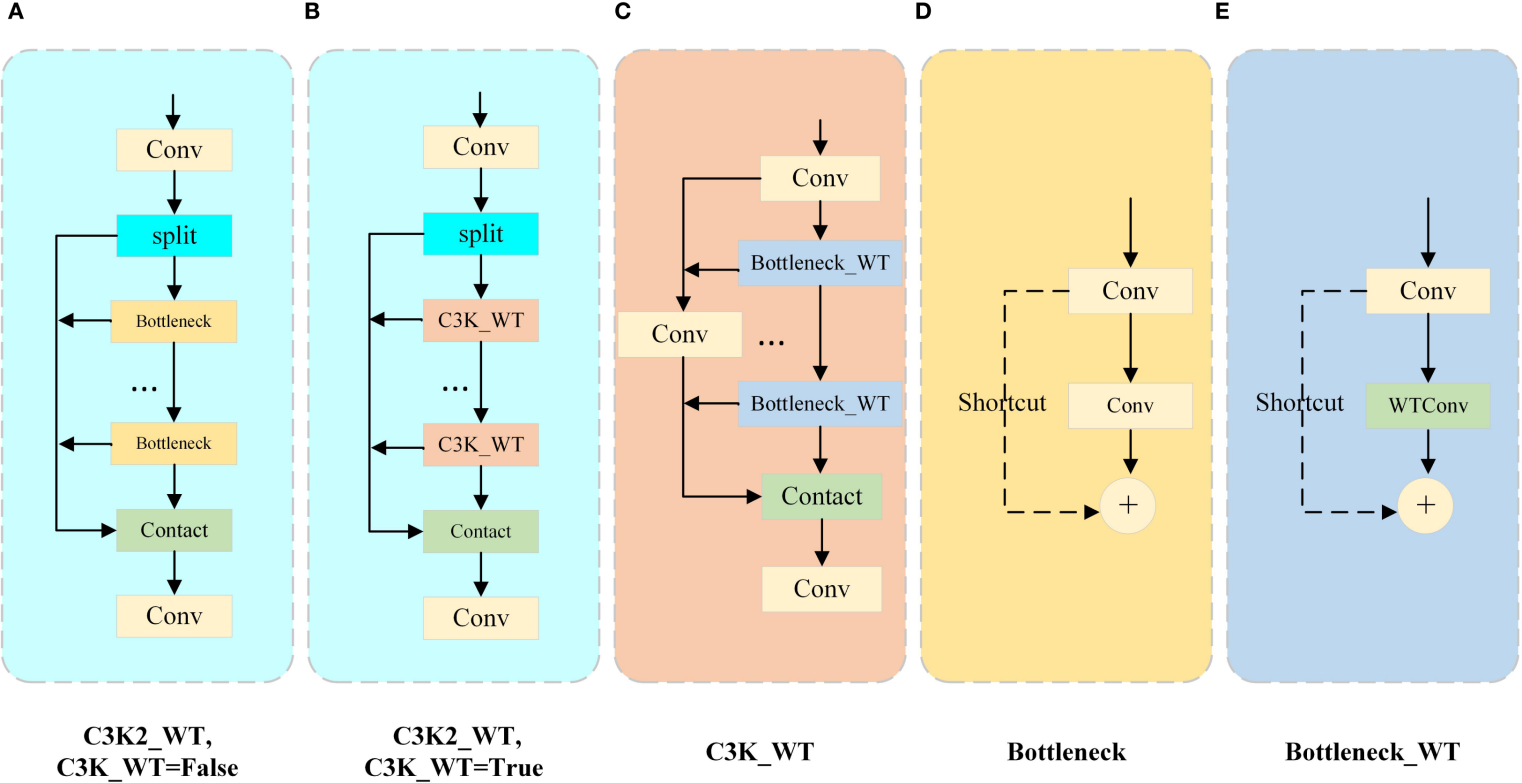
C3K2_WT module structure. **(A)** C3K2_WT structure when C3K_WT is True; **(B)** C3K2_WT structure when C3K_WT is False; **(C)** C3K_WT structure; **(D)** Bottleneck structure; **(E)** Bottleneck_WT structure.

In this study, the WTConv module is integrated into the C3K2 structure, resulting in a novel structure named C3K2_WT, as illustrated in **Figure 5**. Firstly, inspired by the principles of the Wavelet Transform (WT), the standard convolutional layers within the Bottleneck block are replaced with WTConv modules. This leads to the design of a new Bottleneck_WT module (**Figure 5E**). This module effectively enlarges the receptive field and enables the extraction of more discriminative features, particularly for pests with highly similar textures and colors, thereby enhancing the model’s feature extraction capability. Secondly, leveraging the branching characteristics of the C3K2 structure, we further embed the Bottleneck_WT module specifically into the C3K block. This replaces the original Bottleneck components and forms a new C3K_WT structure (**Figure 5C**). Meanwhile, to maintain model flexibility and adaptability, the original Bottleneck block is also retained (**Figure 5D**). Ultimately, the new C3K2_WT structure is constructed(**Figures 5A, B**). This design enhances the model’s capacity for fine-grained feature extraction and detection accuracy for Scolytinae pests detection, while also effectively reducing the number of parameters.

### 2.5 SE attention mechanism module

The traditional YOLO network architecture directly transmits the output pest feature maps to the detection head for pest detection after feature fusion. However, in this process, the importance of channels is not effectively distinguished. So the key feature information of pests is not fully utilized, which directly affects the accuracy of the model pest detection.

**Figure 6:**
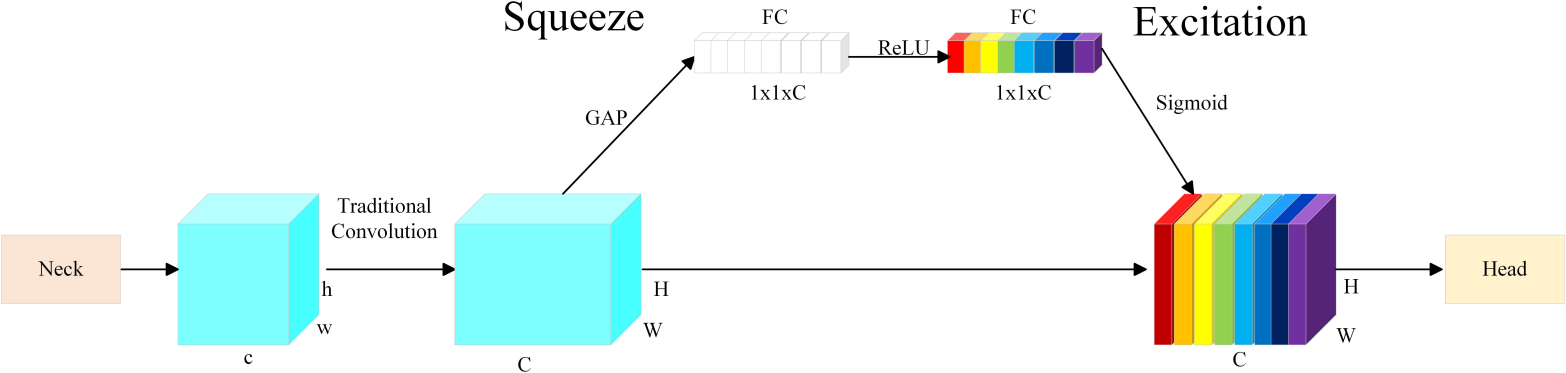
Schematic of the SE attention mechanism.

Considering the high morphological similarity between Scolytinae pests species, which causes issues such as false positives and false negatives during the detection process. We introduce targeted optimizations to the YOLO network architecture to enhance the model’s ability to detect Scolytinae pests. Specifically, the SE(Squeeze and Excitation) (32) attention mechanism is integrated between the Neck and Head networks. This module utilizes the Squeeze operation to extract global contextual information, and then the Excitation operation adaptively learns the importance weights of each channel. This significantly improves the model’s responsiveness to discriminative features in pest images, thereby enhancing its ability to distinguish between categories. As a result, the detection head can focus more effectively on the critical regions of the pests, improving the model’s sensitivity to fine-grained differences between Scolytinae pests species. This enhancement significantly boosts the model’s capability to differentiate between pests in complex backgrounds and highly similar species, thereby improving overall detection accuracy and robustness. The structure is illustrated in **Figure 6**.

## 3 Results

### 3.1 Experimental design

#### 3.1.1 Experimental setup and hyperparameter configuration

This experiment uses PyTorch as the deep learning framework, with the detailed experimental environment shown in **Table 1**. To optimize model training, we combine cosine annealing and early stopping strategies. Cosine annealing dynamically adjusts the learning rate, gradually decreasing it during training to help the model fine-tune parameters in the later stages and avoid the issue of local minima. At the same time, the early stopping strategy monitors the validation loss to prevent overfitting. Training will be stopped early if the validation loss does not significantly converge within 100 consecutive epochs, thus reducing training time and avoiding overfitting. The combination of these two strategies optimizes the model training process and ensures the best performance on the validation set. During training, the batch size is set to 32, the number of epochs is set to 300, the initial learning rate is set to 0.01, weight decay is set to 0.0005, and the momentum factor is set to 0.937. To prevent getting stuck in local optima, the SGD optimizer is used in this experiment.

**Table 1:** Hardware and software environment.

Configuration	Value
CPU	18 vCPU AMD EPYC 9754
GPU	NVIDIA GeForce RTX 3090(24GB)
CUDA	12.1
Deep learning framework	Pytorch 2.1.0
Programming Language	Python 3.10
Operating System	Ubuntu 22.04

#### 3.1.2 Evaluation metrics

To achieve a balance between detection accuracy and model complexity in the Scolytinae pests detection task. This study employs Precision(P), Recall (R), F1 score and mean Average Precision (mAP) as the primary evaluation metrics for assessing the detection performance of the proposed model. Meanwhile, the model complexity is measured in terms of parameters, and GFLOPs. The corresponding calculation formulas are shown in Equations 9-14:


(9) $$Precision=\frac{TP}{TP+FP}\times 100\%$$

(10) $$Recall=\frac{TP}{TP+FN}\times 100\%$$

(11) $$F1=\frac{2\times Precision\times Recall}{Precision+Recall}\times 100\%$$

In Equations 9 and 10: TP (True Positive) refers to the number of correctly detected pests; FN(False Negative) denotes the number of pests that were not correctly detected; TN (True Negative) indicates the number of non-pest instances that were correctly classified as non-pests; FP (False Positive) refers to the number of non-pest instances that were incorrectly detected as pests. In Equation 11, the F1 score is defined as the harmonic mean of Precision and Recall, which comprehensively evaluates the trade-off between accuracy and completeness of the model.


(12) $$mAP=\frac{1}{n}\sum_{1}^{n}P_{i}=\frac{1}{n}\left(P_{1}+P_{2}+\cdots +P_{n}\right)$$

(13) $$mAP@50\sim 95=\frac{1}{c}\sum_{k=1}^{c}mAP@50_{k}$$

(14) $$mAP@50\sim 95=\frac{1}{10}\left(mAP@50+mAP@55+\cdots mAP@95\right)$$

In Equations 12-14: mAP@0.5 represents the mean Average Precision when the Intersection over Union(IoU) threshold is set to 0.5. mAP@0.5~0.95 refers to the average of mAP values calculated at multiple IoU thresholds ranging from 0.5 to 0.95 with a step size of 0.05. This metric adopts a stricter and more comprehensive evaluation standard, offering a more accurate reflection of the model’s overall detection performance.

#### 3.1.3 Model architecture and parameter details

GIWT-YOLO is primarily used for the detection of Scolytinae pests. First, the input image is resized to 640×640, and Mosaic data augmentation is applied to improve the model’s generalization ability. Next, the preprocessed image is input into the backbone network for feature extraction, capturing multi-scale pest features. The extracted features then enter the neck network, where features from different scales are further fused to enhance the detection capability for small pests. Finally, the fused feature maps are refined using the SE attention mechanism for channel weight adjustment. The detection head then performs object classification and bounding box regression, producing the final detection results. The detailed network architecture of GIWT-YOLO is shown in **Table 2**.

**Table 2:** Detailed Parameters of GIWT-YOLO Network.

Structure	Module	Output Size	Parameters
Backbone	Conv	160×160	928
	GIConv	80×80	11296
	C3K2_WT(*P2*)	80×80	26080
	GIConv	40×40	81728
	C3K2_WT(*P3*)*	40×40	103360
	GIConv	20×20	326272
	C3K2_WT(*P4*)*	20×20	293632
	GIConv	10×10	713984
	C3K2_WT	10×10	1127936
	SPPF	$\{\begin{array}{c} \text{maxpool},5\times 5\\ \text{maxpool},9\times 9\\ \text{maxpool},13\times 13 \end{array}$	656896
	C2PSA(*P5*)*		990976
Neck	*P3*_in	P3→40×40	
	*P4*_in	P4→20×20	
	*P5*_in	P5→10×10	
Head	$\to SE\to \{\begin{array}{c} CIoU\\ CLSLoss \end{array}$		

* The extracted features from layers *P3*, *P4*, and *P5* of the backbone are passed to the Neck network for further processing and feature fusion.

### 3.2 Result and analysis

#### 3.2.1 Performance comparison and analysis of various object detection models

Since GIWT-YOLO is developed upon the YOLO11 architecture, comparing it with earlier versions of YOLO provides a direct and meaningful reference to assess the effectiveness of the proposed improvements. To comprehensively evaluate the detection performance of the GIWT-YOLO model, we conducted comparative experiments using the same experimental setting and training hyperparameters with YOLOv5s (11), YOLOv8s (14), YOLOv9s (16), YOLOv10s (15), RT-DETR-l (33), RT-DETR-resnet50 (33), and the baseline model YOLOv11s (13). The experimental results are shown in **Table 3**.

**Table 3:** The performance comparison of different object detection models.

Model	Precision (%)	Recall (%)	F1-Score (%)	mAP@50 (%)	mAP@50~95 (%)	GFLOPs (G)	Parameters (M)
RT-DETR-l	82.0	77.0	79.4	74.9	53.5	108.0	32.8
RT-DETR-resnet50	82.7	77.0	79.8	75.7	53.8	130.5	42.8
YOLOv5s	78.4	78.7	78.6	84.4	61.6	19.0	7.8
YOLOv8s	79.4	82.2	80.8	85.7	60.1	23.6	9.8
YOLOv9s	79.8	79.4	79.6	84.0	62.4	22.7	**6.3**
YOLOv10s	77.9	82.0	79.9	85.0	61.2	24.8	8.1
YOLOv11s	82.5	79.8	81.1	84.7	60.3	21.6	9.4
GIWT-YOLO	**84.7**	**82.2**	**83.4**	**88.7**	**63.4**	**18.7**	8.4

Bold values represent the best experimental results compared to other models.

The main performance of GIWT-YOLO surpasses that of YOLOv5s, YOLOv8s, YOLOv9s, YOLOv10s, RT-DETR-l, RT-DETR-ResNet50, and YOLOv11s models. Specifically, it achieves a Precision of 84.7%, Recall of 82.2%, F1-Score of 83.4%, mAP@50 of 88.7%, and mAP@50~95 of 63.4%. The model’s GFLOPs and Parameters are only 18.7G and 8.4M, respectively, achieving a balance between detection accuracy and model lightweighting.

RT-DETR-l and RT-DETR-resnet50 are Transformer-based (34) object detection models, but their model sizes are relatively large, with model complexity and parameter counts significantly exceeding those of other detection algorithms. RT-DETR-l and RT-DETR-resnet50 have similar complexity and detection accuracy. The GFLOPs and Parameters of RT-DETR-Resnet50 are as high as 130.5G and 42.8M, approximately 7 times and 5 times greater than those of GIWT-YOLO, respectively. However, GIWT-YOLO outperforms RT-DETR-resnet50 by 2% in accuracy, 13% in mAP@50, and 9.6% in mAP@50~95. Overall, the performance of the GIWT-YOLO model far exceeds that of RT-DETR-l and RT-DETR-resnet50, achieving a balance between detection accuracy and model lightweighting.

**Figure 7:**
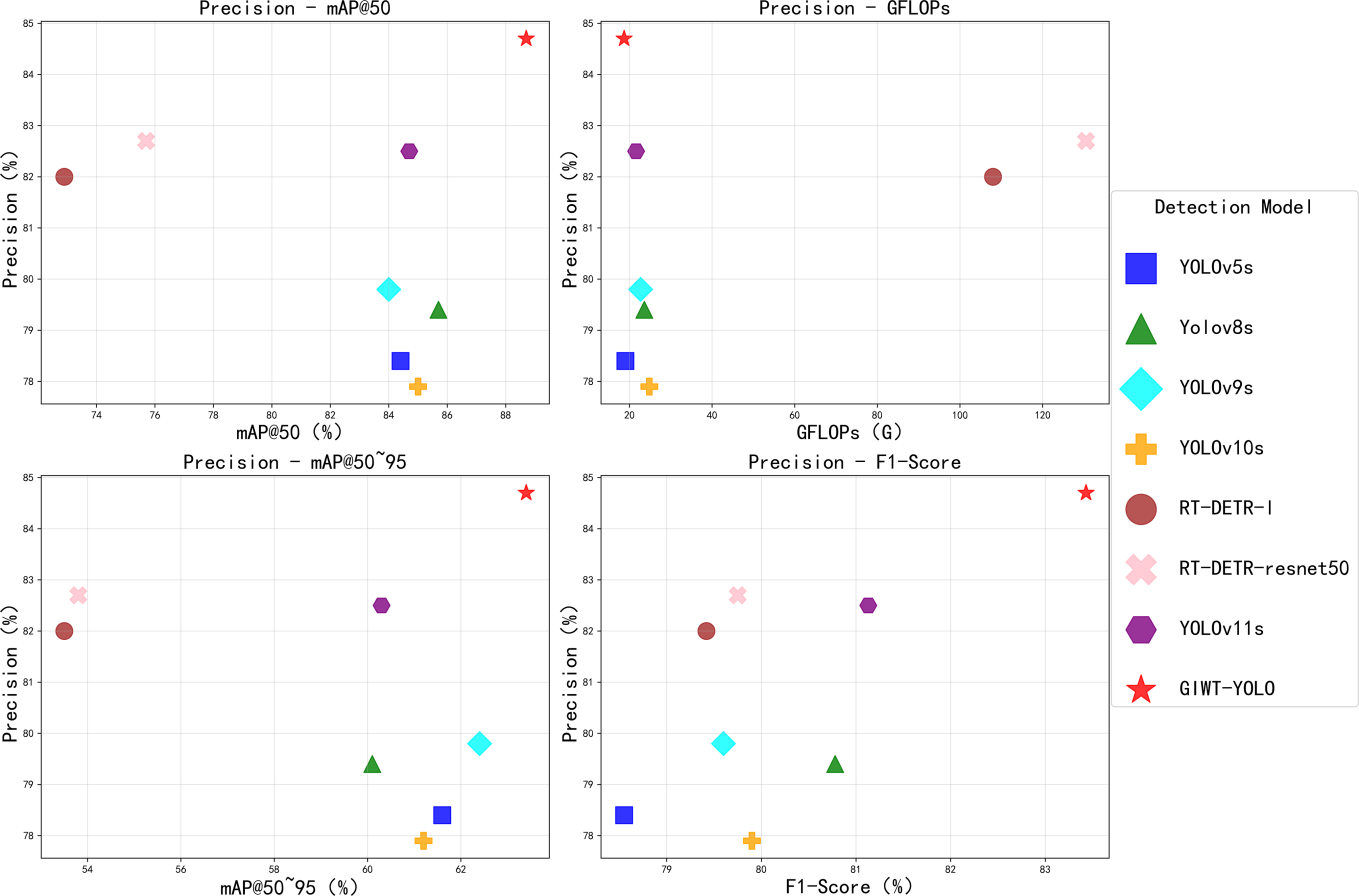
Scatter plot of the performance of different models.

Compared to the baseline model YOLOv11s, the GIWT-YOLO model improved by 2.2%, 2.4%, 2.3%, 4%, and 3.1% in Precision, Recall, F1-Score, mAP@50, and mAP@50~95, respectively, while reducing model GFLOPs and Parameters by 13.4% and 11.3%, respectively. Additionally, although YOLOv8s, YOLOv10s, and GIWT-YOLO have similar parameters, GIWT-YOLO significantly outperforms the other models in Precision, Recall, F1-Score, and mAP, fully demonstrating the effectiveness and superiority of the proposed improvements. These experimental results show that GIWT-YOLO achieves higher detection accuracy and stronger generalization ability while maintaining low GFLOPs. The model, designed to address the significant size differences among Scolytinae pests, adopts a multi-scale feature extraction strategy. This enables it to effectively capture features of pests of various sizes. To further comprehensively showcase the superiority of GIWT-YOLO’s performance, we present a comparison of Precision with mAP@50, mAP@50~95, GFLOPs, and F1-Score, as shown in **Figure 7**.

#### 3.2.2 Ablation experiment

To evaluate the performance of the GIConv, C3K2_WT, and SEAttention modules integrated into the model, we conducted ablation experiments on the Scolytinae pests dataset from Beijing Forestry University. Starting with the YOLOv11s model, we progressively added each improvement module. Model1 replaced the standard convolution modules in the backbone network of YOLOv11s with the GIConv module. Model2 integrated the C3K2_WT module into the original YOLOv11s model. Model3 combined the GIConv module and the C3K2_WT module in the original model. Finally, based on Model3, we integrated the SEAttention module was integrated into both the Neck and Head networks, forming the final GIWT-YOLO model.

As shown in **Table 4**, based on the YOLOv11s model, Model1 introduced the innovative GIConv module. The training results demonstrate that the model achieved improvements of 1.8%, 2.6%, and 2.0% in Recall, mAP@50, and mAP@50~95, respectively, while the parameter count and GFOLPs decreased by 8.5% and 11.6%. This indicates that by addressing the size differences of Scolytinae pests in the images, the model effectively captures more relevant pest features through multi-scale convolution, resulting in more accurate detection and a reduced false negative rate. Model2, built upon the baseline YOLOv11s model, integrated the C3K2_WT module. By effectively expanding the receptive field, it enhanced the detection accuracy of pests with similar textures and colors. This improvement led to mAP@50 and mAP@50~95 values of 85.9% and 63.4%, respectively, which are 1.2% and 3.1% higher than those of the baseline model. Model3, based on Model1, integrated the C3K2_WT module. Through multi-scale convolution and expanded receptive fields, this model improved Scolytinae pests detection accuracy, with increases of 4.2%, 0.4%, and 0.9% in Precision, mAP@50, and mAP@50~95, respectively. Finally, compared to Model3, GIWT-YOLO enhanced the feature extraction capability by introducing the SE module. This strengthened the model’s focus on key feature regions, allowing it to more accurately capture the differences between different Scolytinae pests. As a result, Recall increased by 1.2%, mAP@50 increased by 1.0%, and mAP@50~95 increased by 0.2%. However, due to the impact of channel weighting, the model placed greater emphasis on key feature regions while neglecting some feature information, leading to a slight decrease of 1.6% in Precision. Nevertheless, the improvement in mAP@50~95 indicates enhanced object bounding box regression performance at different IoU thresholds, leading to an overall improvement in detection performance.

**Table 4:** Ablation Experiment of GIWT-YOLO Model.

Model	GIConv	C3K2_WT	SE	Precision (%)	Recall (%)	mAP@50 (%)	mAP@50~95 (%)	GFLOPs (G)	Parameters (M)
YOLOv11s				82.5	79.8	84.7	60.3	21.6	9.4
Model1	✓			82.1	81.6	87.3	62.3	19.1	8.6
Model2		✓		82.1	81.4	85.9	63.4	21.1	9.1
Model3	✓	✓		**86.3**	81.0	87.7	63.2	18.7	**8.3**
GIWT_YOLO	✓	✓	✓	84.7	**82.2**	**88.7**	**63.4**	**18.7**	8.4

Bold values represent the best experimental results compared to other models.

**Figure 8:**
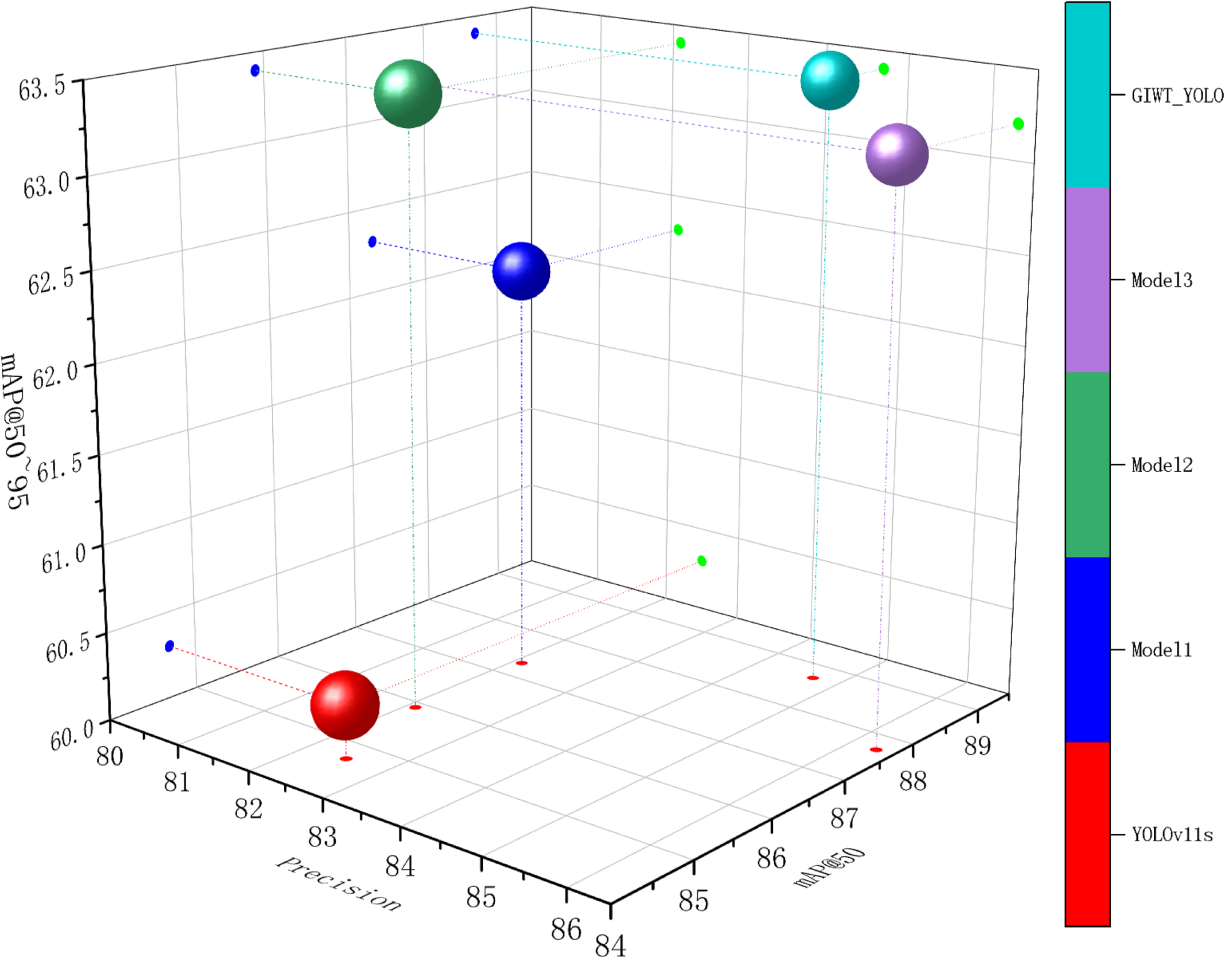
Performance Comparison Among Different Models. Precision as the X-axis, mAP@50 as the Y-axis, and mAP@5095 as the Z-axis. The color in the legend represents different models, and the size of the balls corresponds to GFLOPs.

In summary, compared to the baseline model YOLOv11s, GIWT-YOLO achieves improvements of 2.2%, 2.4%, 4%, and 3.1% in Precision, Recall, mAP@50, and mAP@50~95, respectively. At the same time, the model’s parameters and GFLOPs are reduced by 11.3% and 13.4%, enhancing pest detection accuracy while achieving a lightweight design, enabling efficient detection of differently sized Scolytinae pests under limited computational resources. As shown in **Figure 8**, a comprehensive comparison of different model performances is presented.

#### 3.2.3 Comparison of different lightweight convolutions

**Figure 9:**
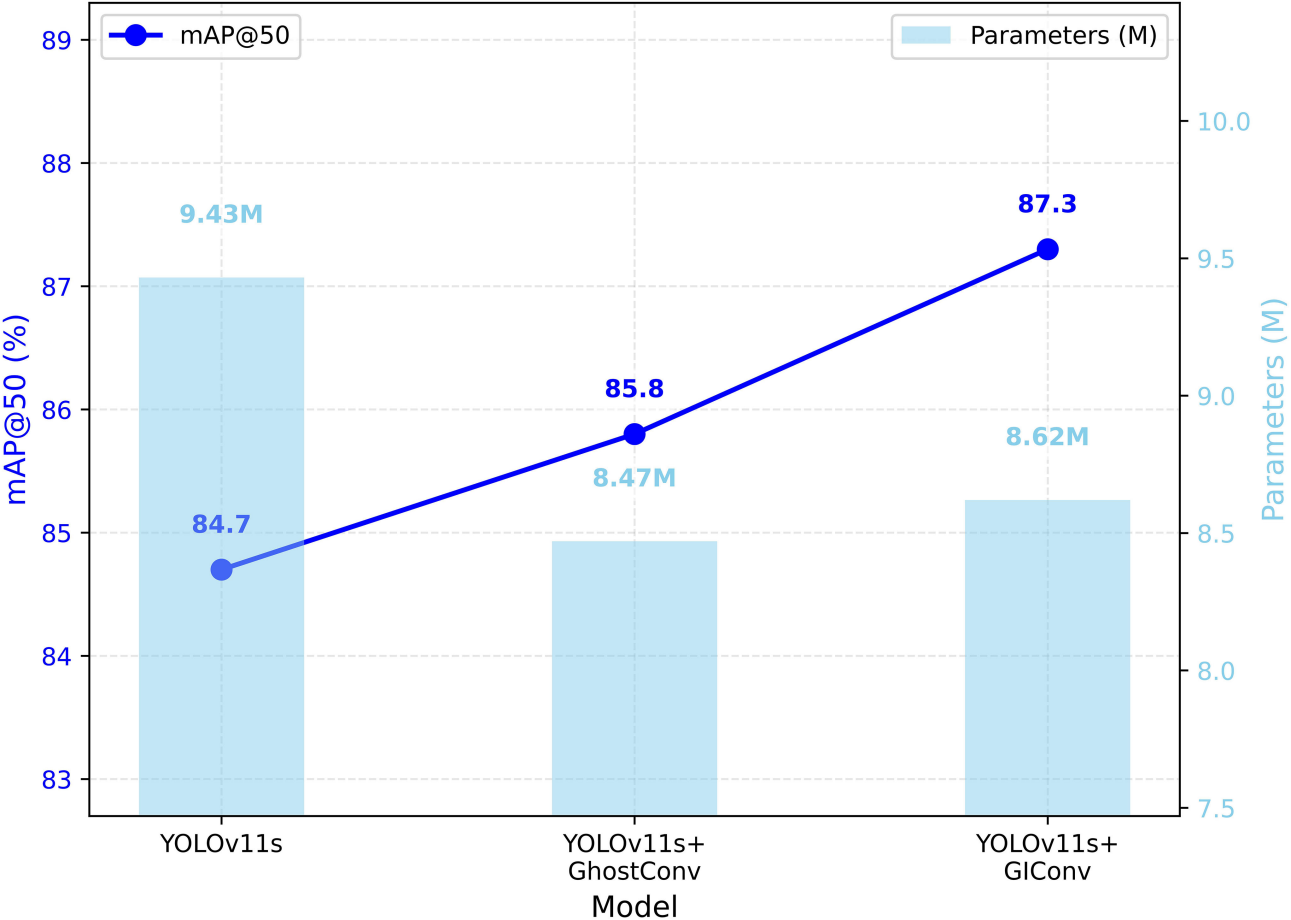
Comparison of different convolutional performance.

When designing GIConv module, we verified its effectiveness through comparative analysis with the standard Convolution and GhostConv (30) modules. As shown in **Table 5**, compared with the GhostConv module, although the amount of GFLOPs is slightly increased by 0.3G and the number of parameters is 0.15M, the improvement in Precision, Recall, F1-Score, mAP@50 and mAP@50~95 indicators is more significant. Compared with standard Convolution, GIConv has significant performance improvement except for a slight decrease of 0.4% in Precision. Overall, the proposed GIConv module demonstrates superior performance. **Figure 9** illustrates the performance curves using different convolution modules.

**Table 5:** Experimental results for the lightweight convolutions.

Model	Precision (%)	Recall (%)	F1-Score (%)	mAP@50 (%)	mAP@50~95 (%)	GFLOPs (G)	Parameters (M)
YOLOv11s	**82.5**	79.8	81.1	84.7	60.3	21.6	9.4
YOLOv11s+GhostConv	80	81.2	80.6	85.8	61.7	**18.8**	**8.5**
YOLOv11s+GIConv	82.1	**81.6**	**81.9**	**87.3**	**62.3**	19.1	8.6

Bold values represent the best experimental results compared to other models.

#### 3.2.4 Comparison of different attention modules

**Figure 10:**
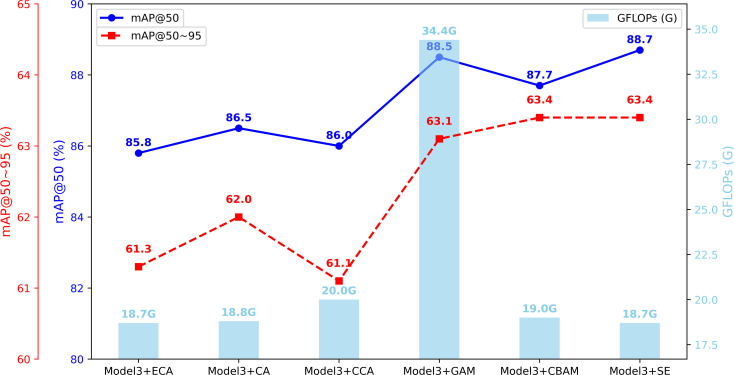
Comparison chart of various attention mechanisms. Model3 integrates GIConv module and C3K2_WT module into YOLOv11s.

To investigate the impact of attention mechanisms on model performance and determine their optimal placement. This study inserts attention modules between the neck network and head network of the model, including SE (32), CBAM (35), CA (36), ECA (37), GAM (38), and CCA (39). A comparative analysis of their performance is conducted. As shown in **Table 6**, among the six attention mechanisms, the SE module achieves the best performance in terms of Precision, F1-Score, mAP@50, and mAP@50–95, while also maintaining the lowest computational complexity. Although the ECA module achieves the highest Recall, it performs the worst in terms of Precision and mAP@50. Overall, the SE module effectively enhances feature representation, improves detection accuracy and stability. It is maintains a low computational cost, making it more advantageous for practical applications. **Figure 10** shows the performance curves of different attention mechanisms.

**Table 6:** Comparison of different attention models’ performance.

Model	Precision (%)	Recall (%)	F1-Score (%)	mAP@50 (%)	mAP@50~95 (%)	GFLOPs (G)	Parameters (M)
Model3+CBAM	84.7	81.4	83.0	87.7	63.4	19.0	8.7
Model3+CA	83.7	80.9	82.3	86.5	62.0	18.8	8.4
Model3+ECA	66.9	**83.5**	74.3	85.8	61.3	18.7	8.3
Model3+GAM	81.2	79.2	80.2	88.5	63.1	34.4	16.9
Model3+CCA	80.6	77.7	79.1	86.0	61.1	20.0	9.0
Model3+SE	**84.7**	82.2	**83.4**	**88.7**	**63.4**	**18.7**	8.4

Bold values represent the best experimental results compared to other models. Model3 integrates GIConv module and C3K2_WT module into YOLOv11s.

We investigated different placement strategies for attention modules within the network to evaluate their effects on detection performance, as shown in **Table 7**. The SE module inserted between neck network and head network compared with the backbone network, it improves the Precision, Recall, F1-Score, mAP@50, and mAP@50~95 by 2.3%, 2.5%, 2.4%, 1.7%, and 1.2%, respectively. This phenomenon is related to the feature requirements at different stages of the network. Inserting the SE module at the backbone network will lead to the squeeze and excitation of low-level features, prematurely affecting the weight of feature channels. This results in the loss of part of feature information and the decline of detection ability.

**Table 7:** Experimental results on the effects of inserting attention modules at different positions.

Model	Embedding position	Precision (%)	Recall (%)	F1-Score (%)	mAP@50 (%)	mAP@50~95 (%)	GFLOPs (G)	Parameters (M)
Model3+SE	Backbone	82.4	79.7	81.0	86	62.2	18.7	8.4
Model3+SE (GIWT-YOLO)	Neck-Head	**84.7**	**82.2**	**83.4**	**88.7**	**63.4**	**18.7**	**8.4**

Bold values represent the best experimental results compared to other models. Model3 integrates GIConv module and C3K2_WT module into YOLOv11s.

#### 3.2.5 Model visualization results

**Figure 11:**
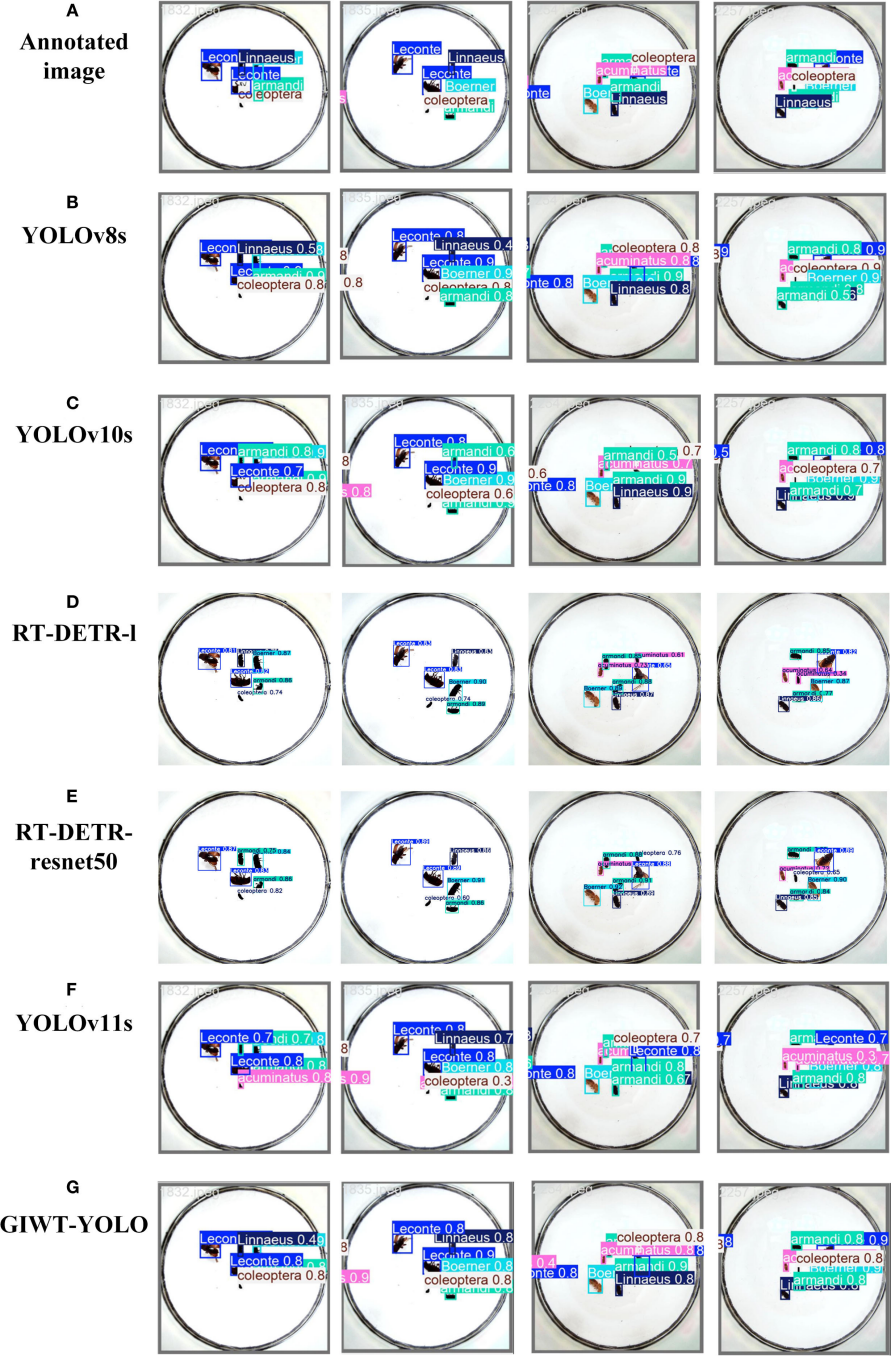
Visualization of different models.

**Figure 12:**
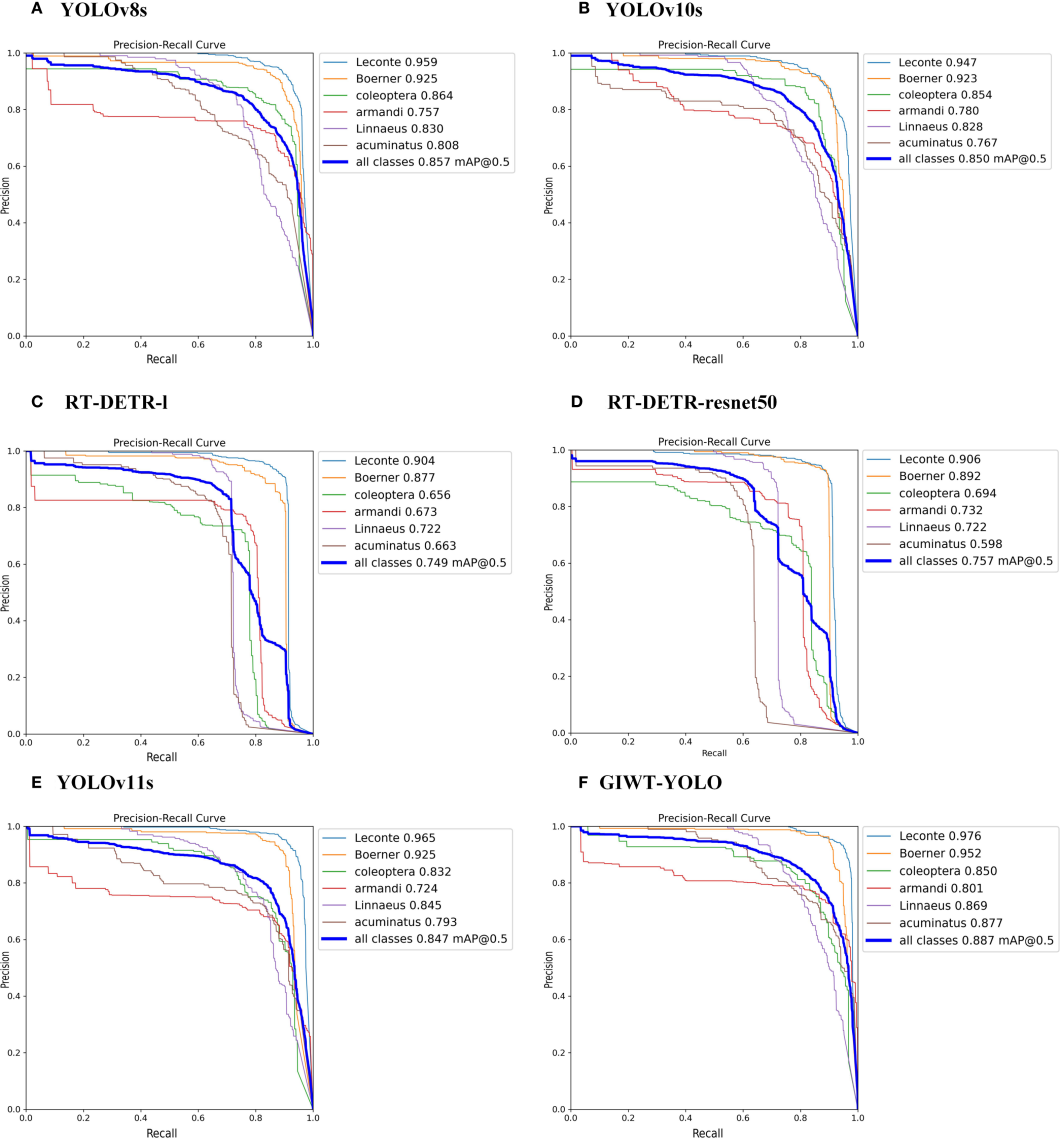
Training PR curves for different models.

To provide a more comprehensive demonstration of the model’s detection performance, this study compares the annotated image, YOLOv8s, YOLOv10s, RT-DETR-l, RT-DETR-resnet50, YOLOv11s, and GIWT-YOLO models. The visualization results are shown in **Figure 11**, and the PR performance curves of each model are presented in **Figure 12**.

Combined with **Figures 10** and **11**, it can be clearly seen that the YOLOv8s (**Figure 11B**, **Figure 12A**) and YOLOv10s (**Figure 11C**, **Figure 12B**) models have weak feature extraction capabilities for small object pests. These models exhibit many false positives and low confidence in bounding boxes, which often result in misclassifying small object pests. For example, incorrectly detecting Linnaeus as belonging to the armandi category. This leads to missed detections for the correct Linnaeus class, causing an increase in false negatives (FN) and a decrease in true positives (TP), thus lowering Recall and the AP for the Linnaeus category. Additionally, misclassifying Linnaeus as armandi leads to an increase in false positives (FP), causing a decrease in Precision for the armandi category and a reduction in AP. On the other hand, RT-DETR-l (**Figure 11D**, **Figure 12C**) and RT-DETR-resnet50 (**Figure 11E**, **Figure 12D**) avoid the NMS (Non-Maximum Suppression) (40) operation. They achieve this by introducing region-aware and global optimization strategies. This effectively prevents redundant detections and the generation of duplicate bounding boxes. However, these models still experience category misclassification, which results in a low mAP.

The YOLOv11s (**Figure 11F**, **Figure 12E**) model exhibits category misclassification along with multiple overlapping bounding boxes. The main reason for this issue is the insufficient detection capability of the model, which lacks focus on small and similarly Scolytinae pests. As a result, some coleoptera are incorrectly classified as acuminatus, leading to AP of only 83.2% and 79.3% for coleoptera and acuminatus, respectively. In contrast, the improved GIWT-YOLO model (**Figure 11G**, **Figure 12F**) utilizes the innovative GIConv module and introduces wavelet transform concepts. It enhances the feature extraction capability of the backbone network through multi-scale convolutions and increased receptive field size. Additionally, an SE attention mechanism is incorporated between the neck network and head network to boost the model’s focus on pest objects, significantly reducing misclassifications and overlapping bounding box issues. As shown in **Table 8**, our method outperforms the state-of-the-art (SOTA) models in terms of AP across multiple pest categories. When compared to the baseline YOLOv11s, the APs for small Scolytinae pests like coleoptera, Linnaeus, armandi, and acuminatus improve by 1.8%, 2.4%, 7.7%, and 8.4%, respectively. The AP for larger Scolytinae pests, such as leconte and Boerner, also show improvements of 1.1% and 2.7%, reaching excellent performances of 97.6% and 95.2%, respectively. In conclusion, the GIWT-YOLO model not only achieves a lightweight design but also significantly outperforms other models in detecting pests of various sizes, particularly small Scolytines pests. This results in a notable enhancement in the accuracy and reliability of forestry pest detection.

**Table 8:** Comparison of average precision (AP) for different pest categories across various models.

Model	AP(%)						
	Small Pests				Larger Pests		
	coleoptera	Linnaeus	armandi	acuminatus	Leconte	Boerner	mean
YOLOv5s	85.2	82.3	72.2	85.2	95.2	89.1	84.4
YOLOv8s	86.4	83.0	75.7	80.8	95.9	92.5	85.7
YOLOv9s	87.3	81.8	69.0	82.4	94.8	88.8	84.0
YOLOv10s	85.4	82.8	78.0	76.7	94.7	92.3	85.0
RT-DETR-l	65.6	72.2	67.3	66.3	90.4	87.7	74.9
RT-DETR-resnet50	69.4	72.2	73.2	59.8	90.6	89.2	75.7
YOLOv11s	83.2	84.5	72.4	79.3	96.5	92.5	84.7
GIWT-YOLO	85.0(↑1.8)	86.9(↑2.4)	80.1(↑7.7)	87.7(↑8.4)	97.6(↑1.1)	95.2(↑2.7)	88.7(↑4.0)

Green upward arrows represent the percentage improvement in AP compared to the baseline model YOLOv11s.

#### 3.2.6 Validating the generalization of the GIWT-YOLO model

To further evaluate the generalization capability of the proposed model, this study employs the publicly available rice pest dataset RP11. Which refines the rice pest subset of IP102 and supplements it with additional images collected via web crawling (41)(https://www.kaggle.com/datasets/dingbiao11/rp11-a-dataset-focus-on-adult-rice-pest, accessed on 22 March 2025).

The dataset comprises 11 categories and a total of 4,559 images. It is randomly divided into training, validation, and test sets in a ratio of 8:1:1. All experimental environments and hyperparameter settings were kept consistent with Section 3.1.1 to ensure fair comparison. The experimental results are shown in **Table 9**. Compared to the YOLOv11s model, GIWT-YOLO demonstrated improvements across all performance metrics. Precision increased by 1.3%, significantly enhancing the detection accuracy for pests of different sizes. Additionally, mAP@50 and mAP@50~95 improved by 0.6% and 0.9%, respectively, indicating more stable detection capability across different IoU thresholds.

**Table 9:** Comparison of generalization verification experimental results.

Model	Precision (%)	Recall (%)	F1-Score (%)	mAP@50 (%)	mAP@50~95 (%)	GFLOPs (G)	Parameters (M)
YOLOv11s	88.4	81	84.5	87.8	75.6	21.6	9.4
GIWT-YOLO	**89.7**	**81.1**	**85.2**	**88.4**	**76.5**	**18.7**	**8.4**

Bold values represent the best experimental results compared to other models.

**Figure 13:**
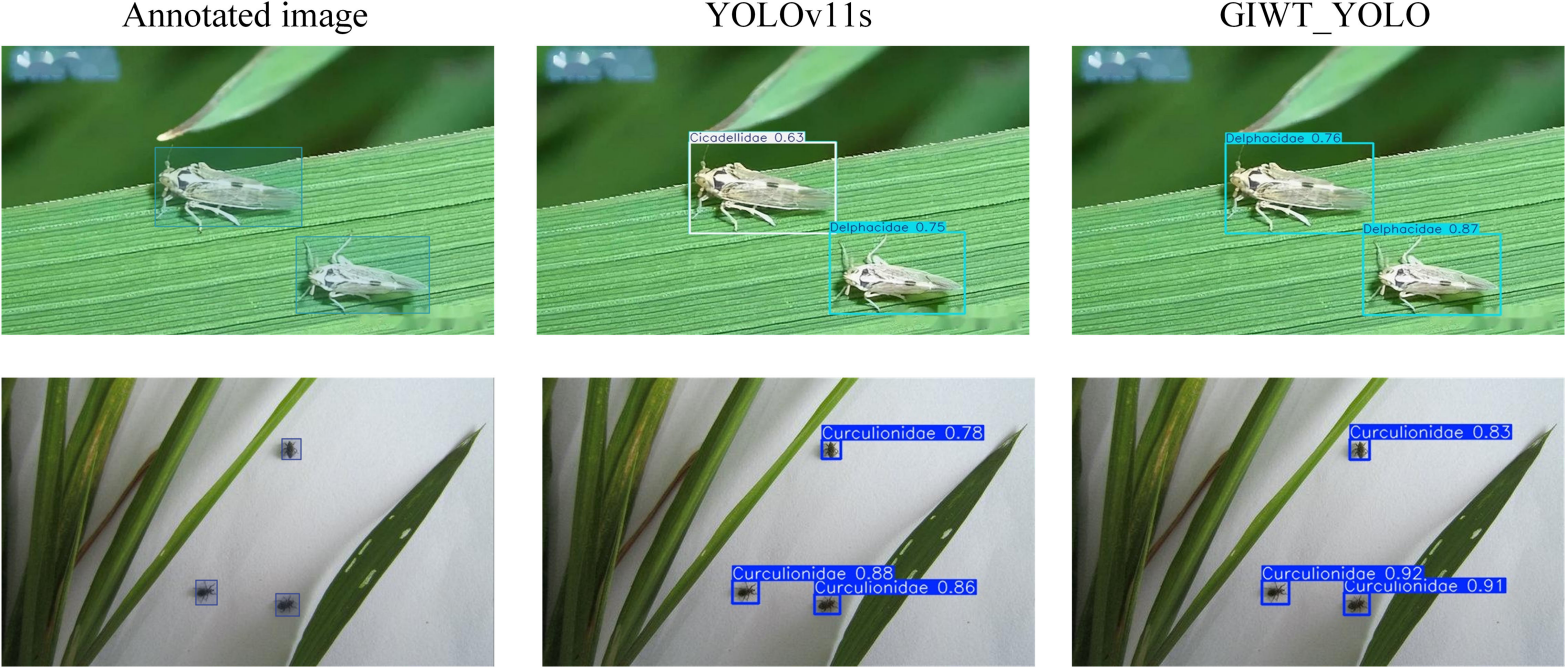
Model Visualization Results.

In real-world field conditions, we visualized the detection results on the RP11 pest dataset. As shown in **Figure 13**, despite challenges such as natural backgrounds and illumination changes, the GIWT-YOLO model was still able to detect most pests with good accuracy, demonstrating strong robustness. In contrast, the baseline YOLOv11s misclassified Delphacidae as Cicadellidae, and its confidence scores were overall lower than those of GIWT-YOLO. These findings highlight the practical applicability of GIWT-YOLO in complex agricultural environments, but it is also evident that complex backgrounds can lead to a decrease in detection performance. In future work, we will place greater emphasis on supplementing datasets collected under real field conditions to further enhance the generalization ability of GIWT-YOLO.

## 4 Discussion

This study proposes a lightweight GIWT-YOLO model based on the improved YOLOv11s, specifically designed for the efficient detection of Scolytinae pests. It provides a valuable technical reference for automated pest monitoring and effectively addresses the detection challenges caused by variations in pest sizes, demonstrating significant application value in forestry pest surveillance and prevention.

Although this study has made progress in the detection of small pests, several limitations remain. Our improved model demonstrates a more balanced performance across different pest sizes. As shown in the PR curves in **Figure 12**, for larger pests, the model maintains a high precision even at around 90% recall. However, for smaller pests, the recall drops to about 85%, accompanied by a faster decline in precision, resulting in more False Negatives and False Positives.

Another limitation lies in the discrepancy between our experimental dataset and real-world field conditions. The Scolytinae pest dataset used in this study was primarily collected in controlled environments with clean backgrounds and relatively simple visual features. However, in actual field conditions, images often contain cluttered and diverse backgrounds such as soil, plant debris, and shadows. Additionally, pests frequently appear in high-density clusters with overlap. As a result, When applied to more complex background environments, our model may experience a decrease in detection accuracy, along with an increase in false positives and false negatives, thereby limiting its effectiveness in real-world applications.

In future work, we plan to enhance the model’s adaptability to complex environmental conditions by supplementing the dataset with more field-acquired imagery. In addition, we aim to further improve the detection accuracy of small pests and reduce False Negatives. Beyond methodological improvements, we also envision deploying the proposed GIWT-YOLO model onto edge devices such as the NVIDIA Jetson Nano. To further reduce model complexity, we plan to explore channel pruning and knowledge distillation techniques. Integrating lightweight detection models with embedded platforms holds great potential for the future development of scalable pest monitoring systems.

## 5 Conclusions

Achieving a balance between accuracy and lightweight design has been challenging for previous studies. In this work, we constructed a GIConv module to enhance multi-scale feature extraction capabilities, particularly for small object pests. This module significantly improves detection performance while reducing the computational cost to only 60% of that of standard convolutional modules, thus achieving a better balance between accuracy and efficiency. Additionally, to address the challenges of detecting pests with similar colors and textures, a WTConv module inspired by wavelet transform was introduced into the C3K2 module. So as to expand the effective receptive field with only a small amount of parameters, improve the detection accuracy of pests with similar color and texture, and further reduce the amount of parameters and calculation. Furthermore, considering the morphological similarities among Scolytinae pests species, an SE attention mechanism was incorporated between the neck and head components of the network. This enhancement increases the model’s focus on key feature regions, enabling more accurate discrimination between similar pest classes. As a result, the proposed GIWT-YOLO model achieved a Precision of 84.7%, Recall of 82.2%, mAP@50 of 88.7%, and mAP@50–95 of 63.4%, with only 18.7 GFLOPs and a final model size of 17.1 MB. Compared to the baseline YOLOv11s model, GIWT-YOLO improves Precision and mAP@50 by 2.2% and 4%, respectively, while reducing the parameter count, computation, and model size by 11.3%, 13.4%, and 10.9%. These results demonstrate that GIWT-YOLO effectively balances detection accuracy and model complexity, offering a more accurate and efficient solution for forestry pest monitoring applications.

## Data Availability

The datasets presented in this study can be found in online repositories. The names of the repository/repositories and accession number(s) can be found in the article/**Supplementary Material**.
