# Supplementary material for: GIWT-YOLO: an efficient multi-scale framework for real-time Scolytinae pests detection
Source: Front Insect Sci. 2025 Sep 26;5:1635439. doi: 10.3389/finsc.2025.1635439 (PMC12511143; doi:10.3389/finsc.2025.1635439)
Supplement: Supplementary file 1 [file SupplementaryFile1.docx]

Supplementary Material

# Supplementary Figures and Tables

## Supplementary

## **Supplementary Figure 1.** Data sample image.

**Supplementary Figure 2.** The proposed GIWT-YOLO algorithm model. The red dashed line represents the added improvement module.


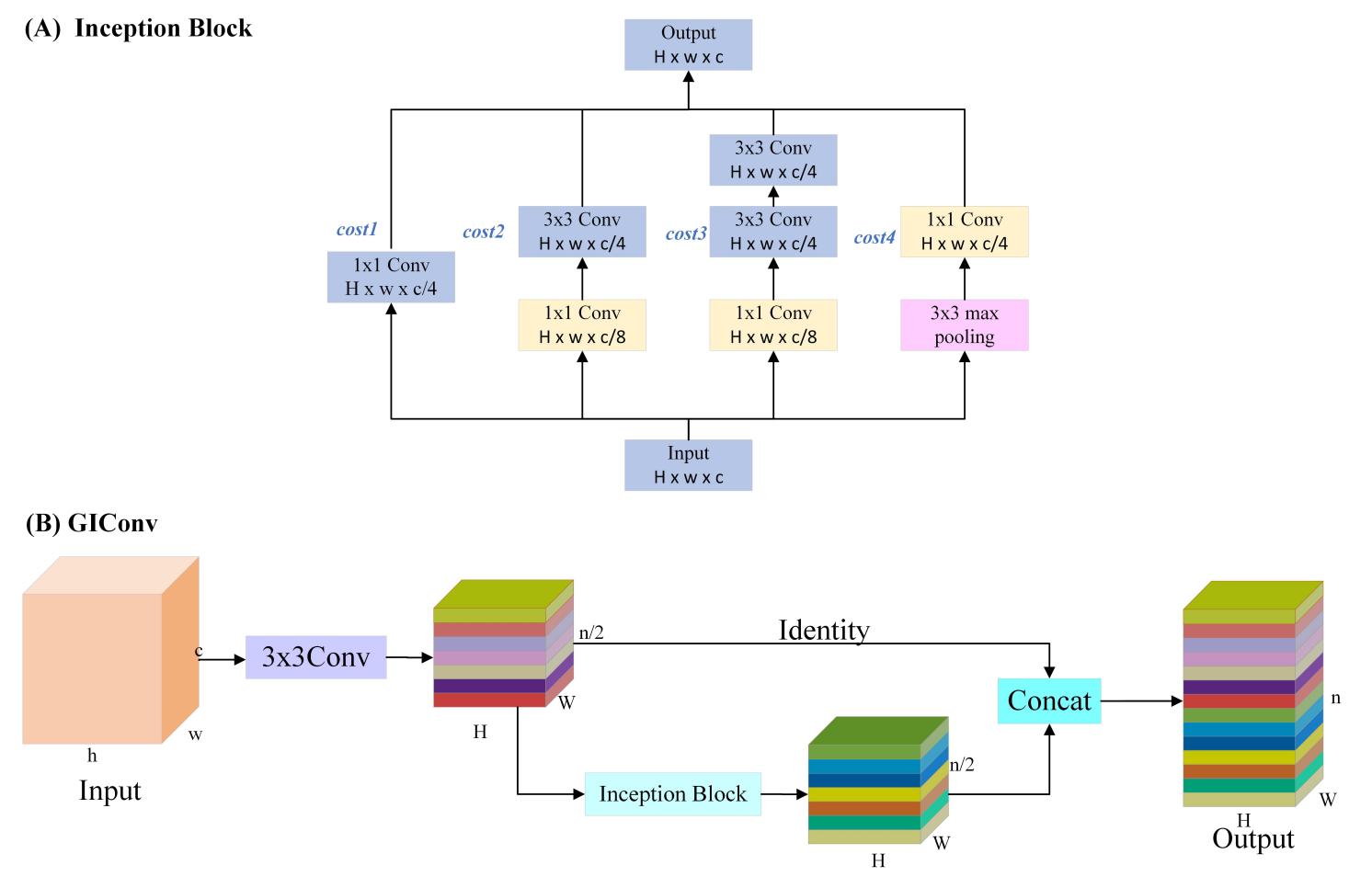


**Supplementary Figure 3.** The structure of the GIConv module. (A) shows a parallel multi-branch structure with multi-scale convolutions; (B) shows the structure of GIConv.


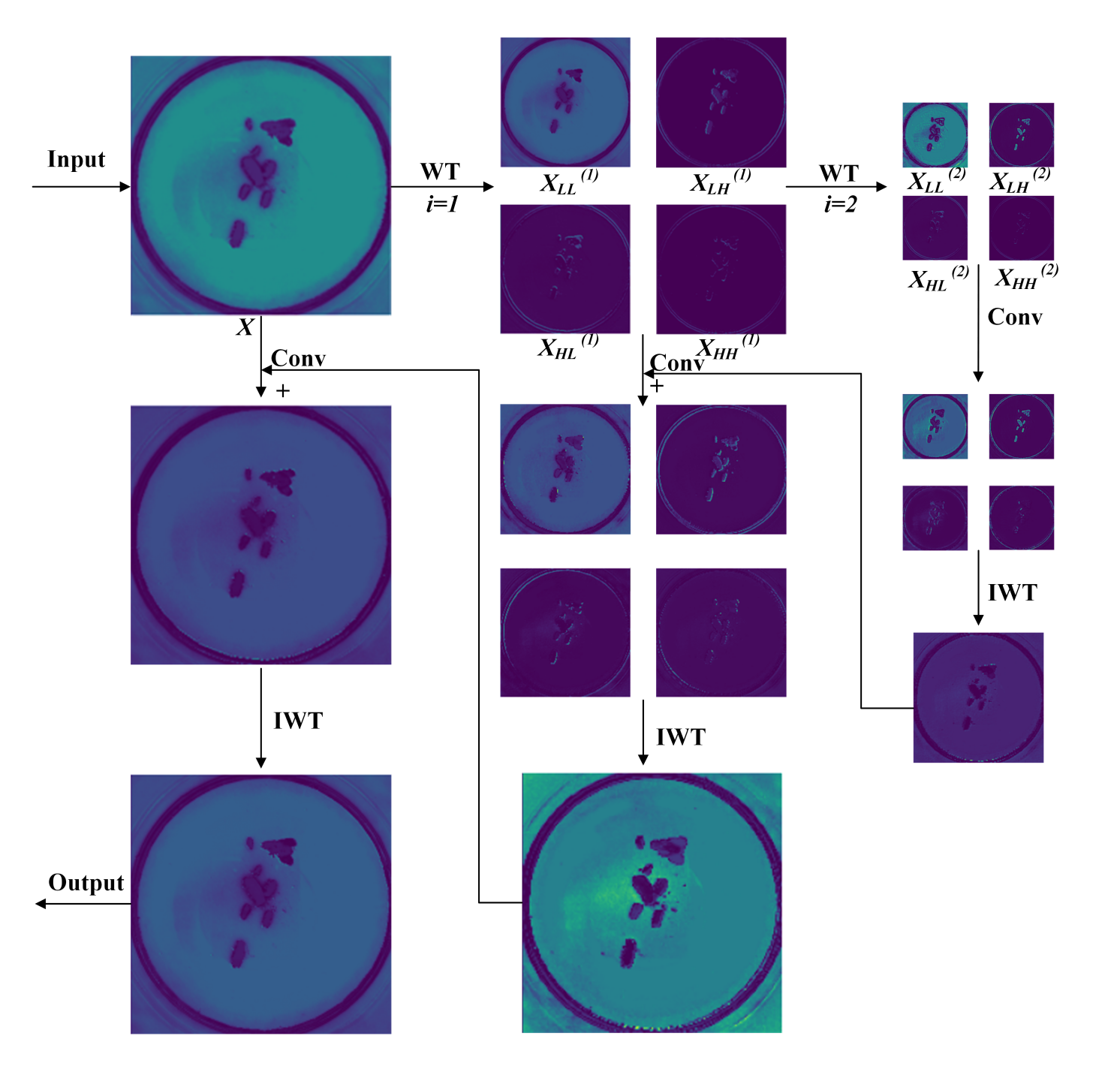


**Supplementary Figure 4.** WTConv module structure. Wavelet Transform (WT) Principle, *X* is the input feature map,$X_{LL}$is the low-frequency component, and$X_{LH}\text{、}X_{HL}\text{、}X_{HH}$is the horizontal, vertical and diagonal high-frequency components. Among them, when *i*=0, $X_{LL}^{\left(0\right)}=X$, *i* represents the current level. "+" represents the Concat operation. IWT represents the Inverse Wavelet Transform operation.


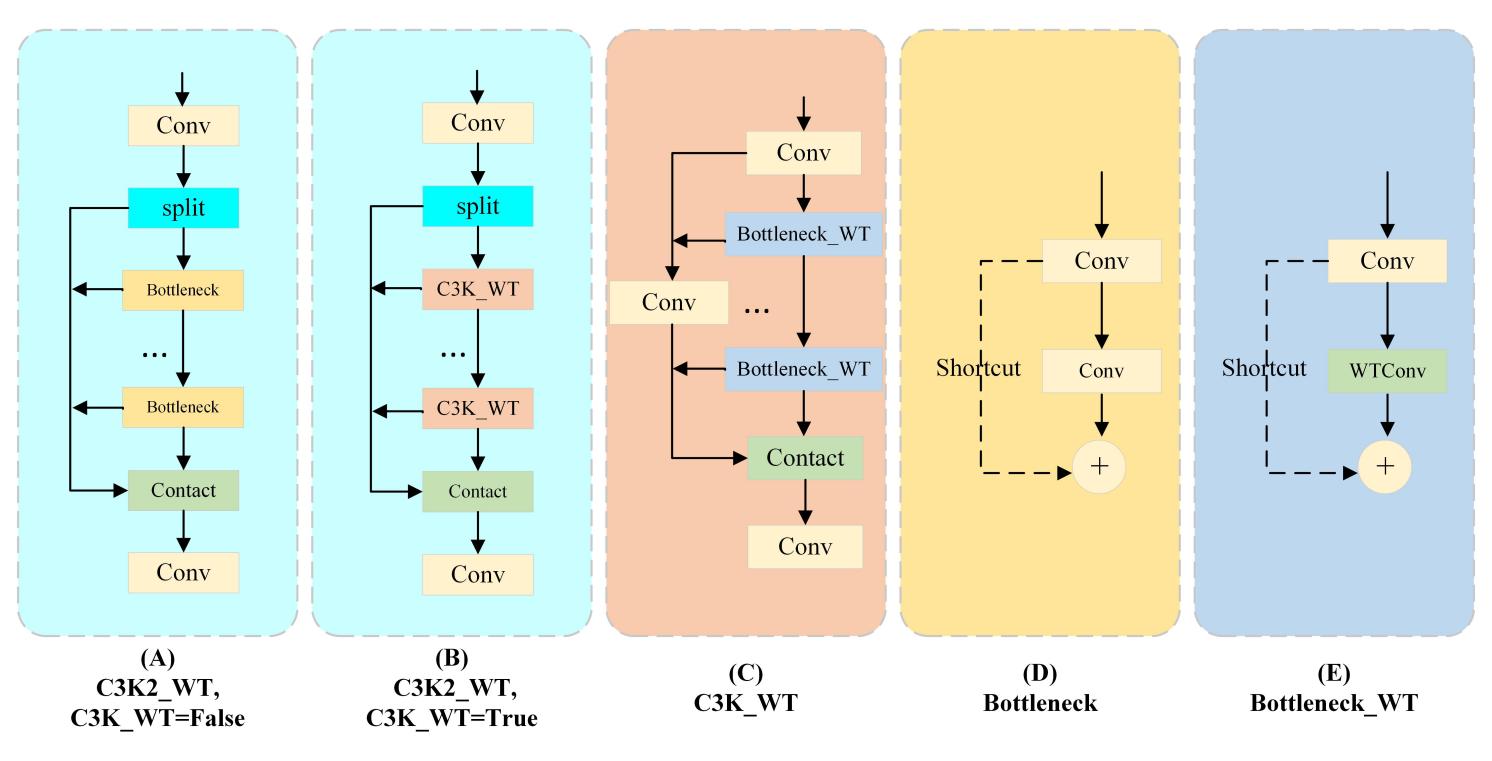


**Supplementary Figure 5.** C3K2_WT module structure. (A) C3K2_WT structure when C3K_WT is True; (B) C3K2_WT structure when C3K_WT is False; (C) C3K_WT structure; (D) Bottleneck structure; (E) Bottleneck_WT structure.


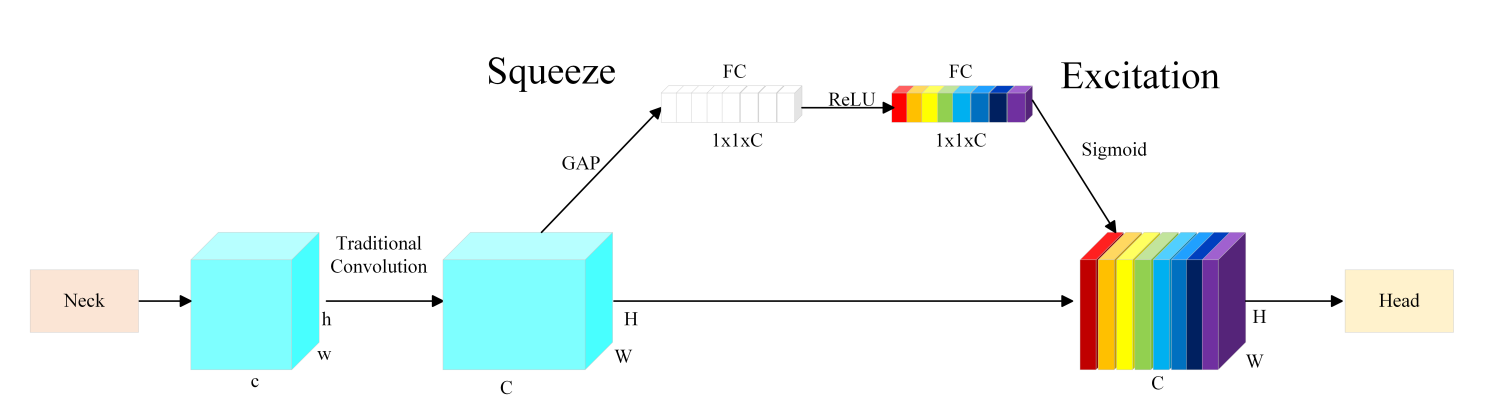


**Supplementary Figure 6.** Schematic of the SE attention mechanism.


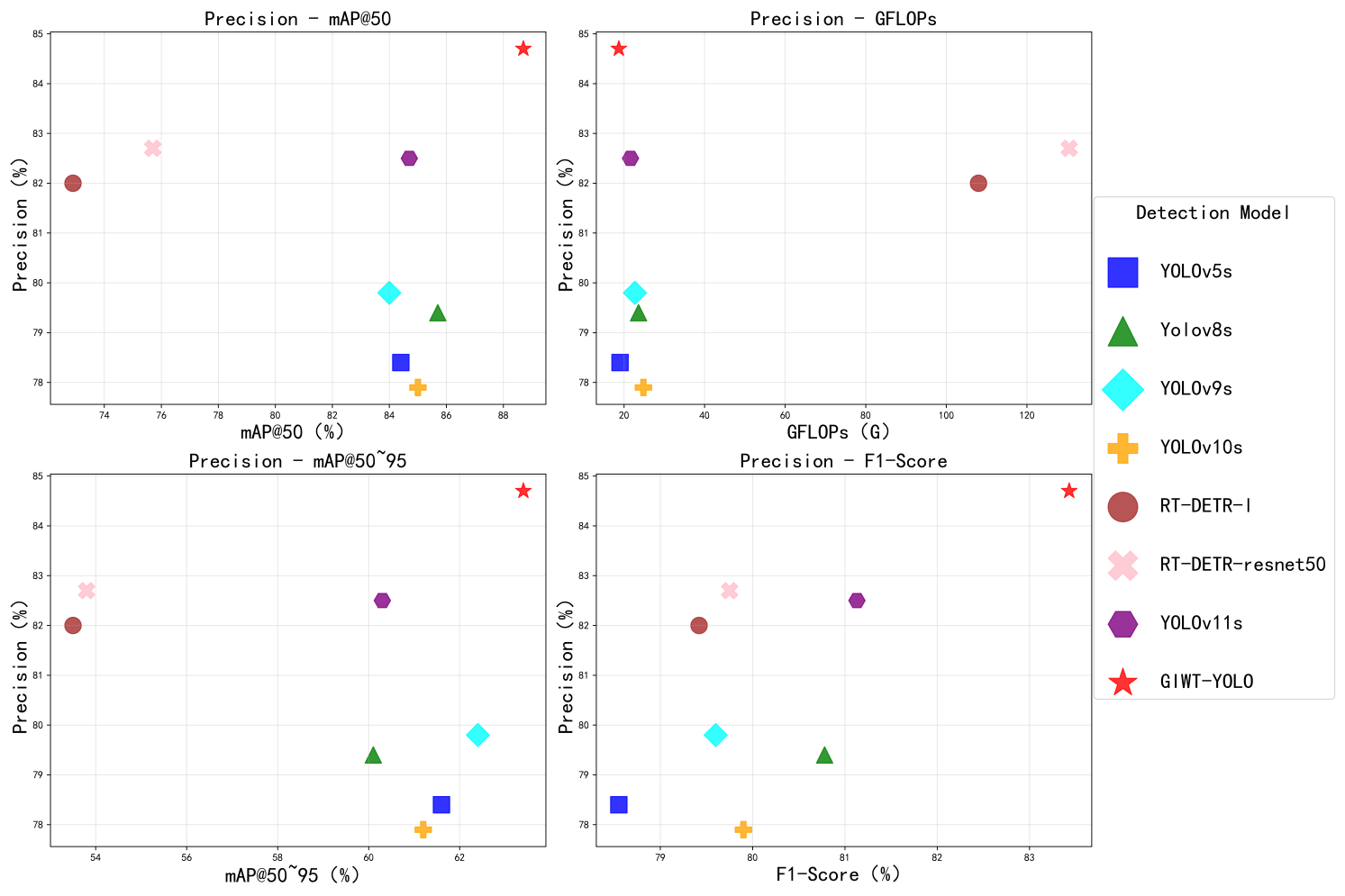


**Supplementary Figure 7.** Scatter plot of the performance of different models.


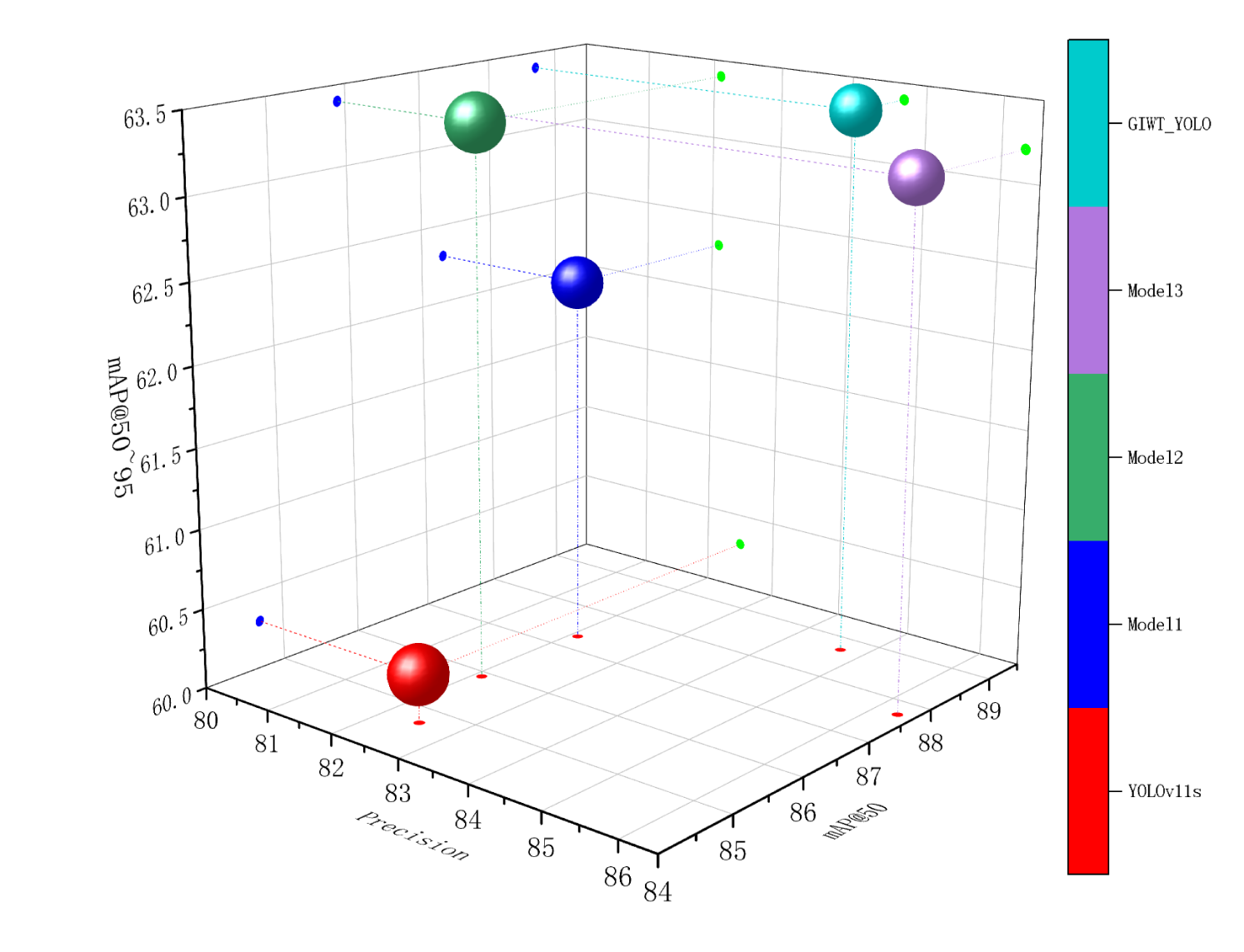


**Supplementary Figure 8.** Performance Comparison Among Different Models. Precision as the X-axis, mAP@50 as the Y-axis, and mAP@5095 as the Z-axis. The color in the legend represents different models, and the size of the balls corresponds to GFLOPs.


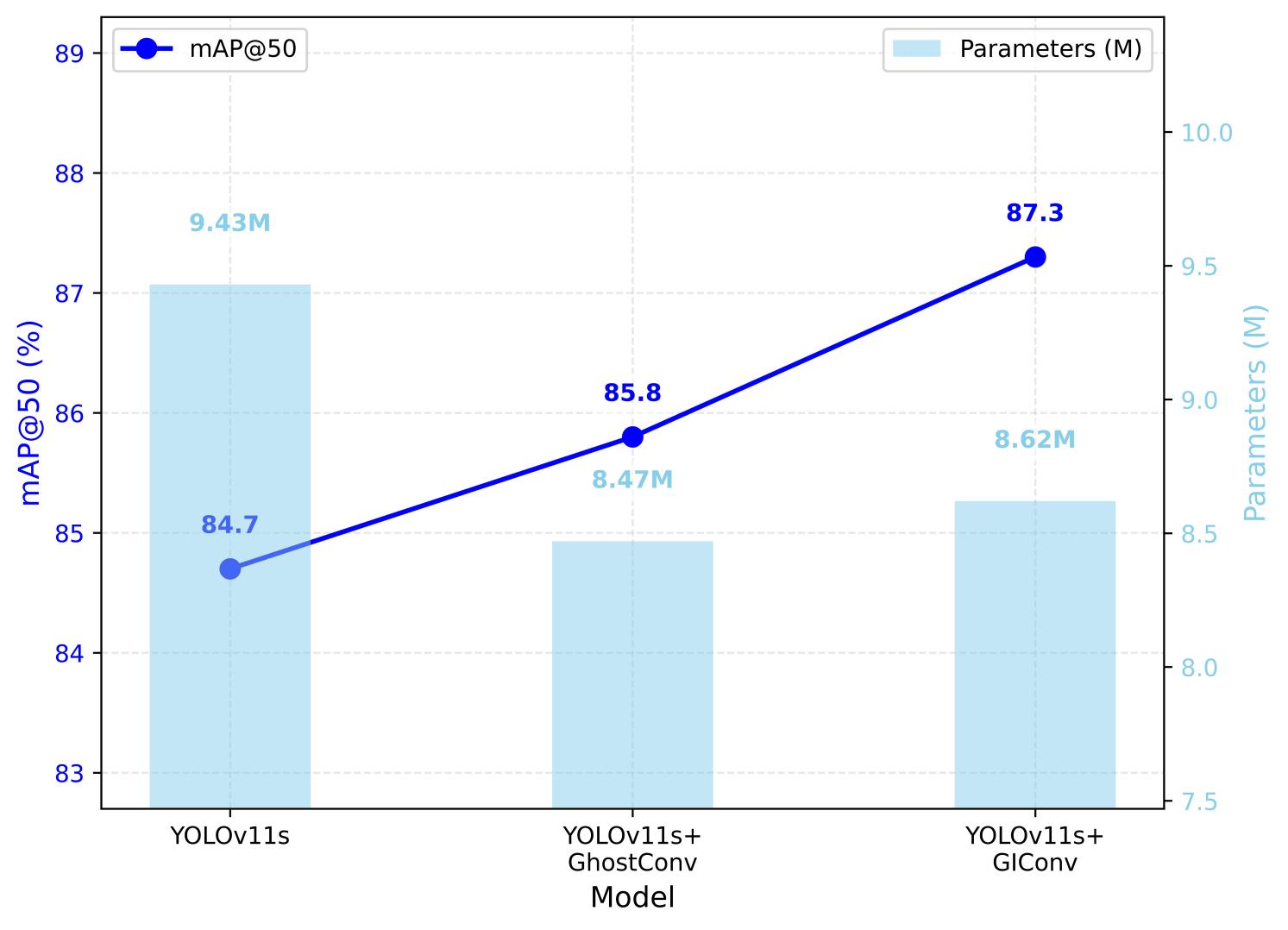


**Supplementary Figure 9.** Comparison of different convolutional performance.


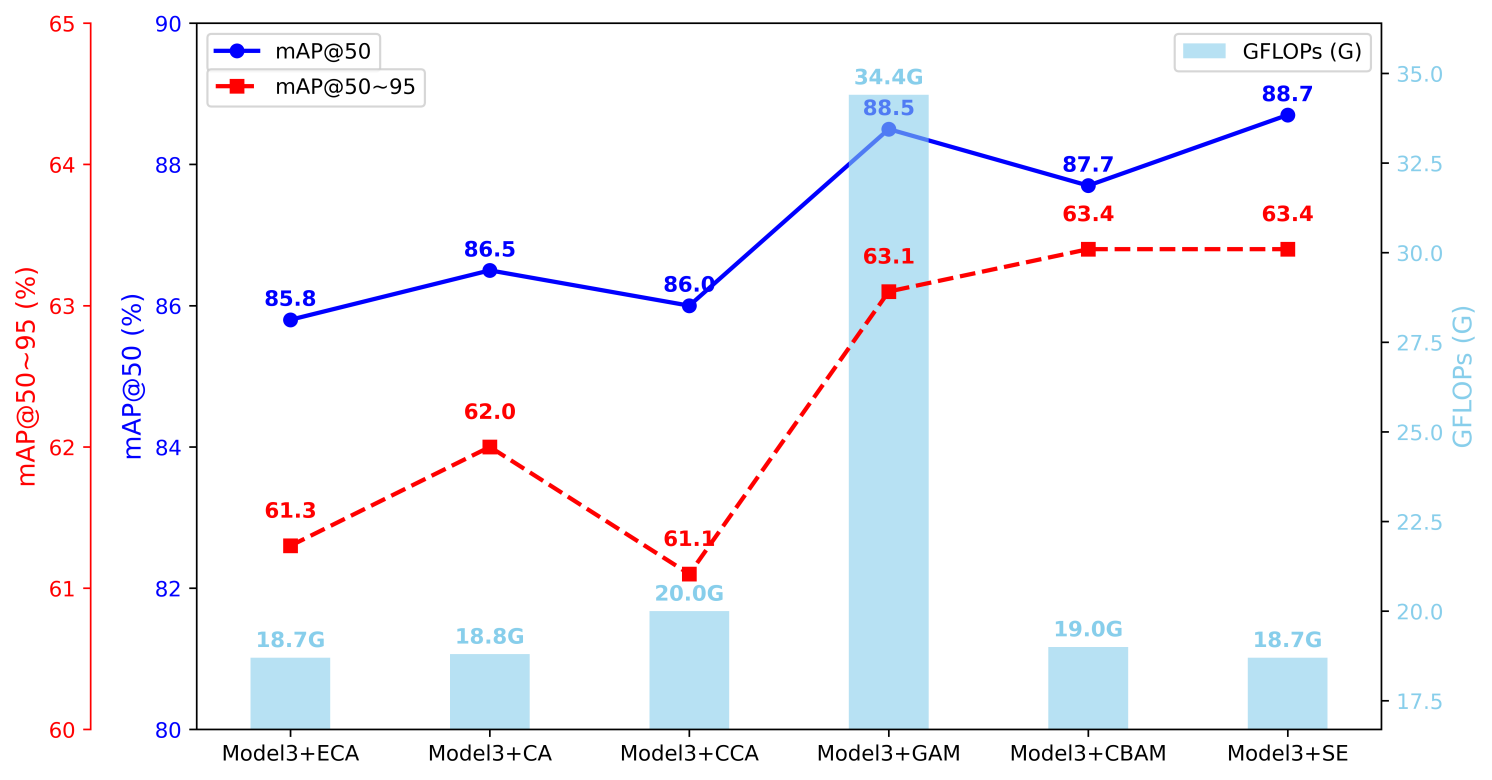


**Supplementary Figure 10.** Comparison chart of various attention mechanisms. Model3 integrates GIConv module and C3K2_WT module into YOLOv11s.

**Supplementary Figure 11.** Visualization of different models.

**Supplementary Figure 12.** Training PR curves for different models.


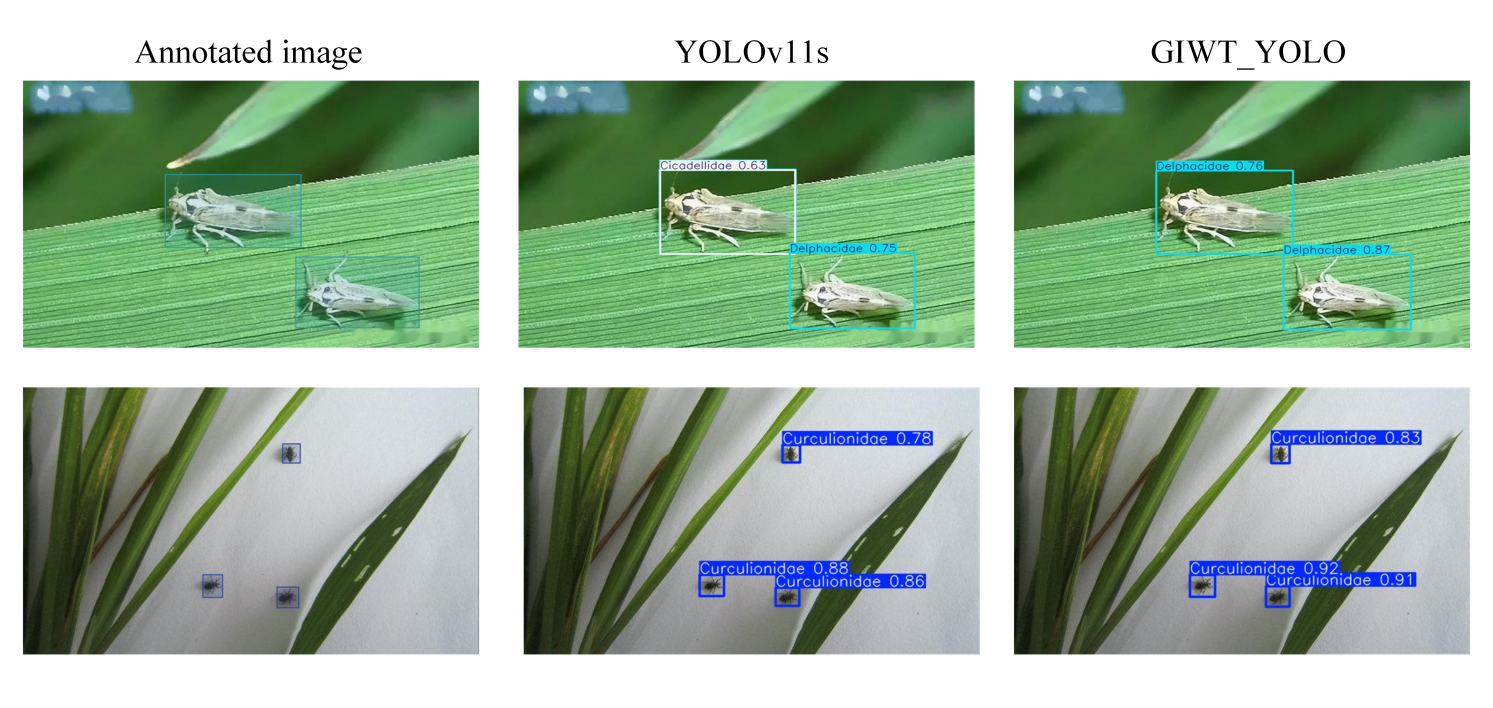


**Supplementary Figure 13.** Model Visualization Results.
